# Designing Maximal Strength in Nanolamellar Eutectic High‐Entropy Alloys

**DOI:** 10.1002/adma.202500149

**Published:** 2025-06-27

**Authors:** Weiming Ji, Shubo Gao, Asker Jarlöv, Xiaojun Shen, Yujia Tian, Mao See Wu, Huajian Gao, Kun Zhou

**Affiliations:** ^1^ School of Mechanical and Aerospace Engineering Nanyang Technological University 50 Nanyang Avenue Singapore 639798 Singapore; ^2^ School of Electrical and Electronic Engineering Nanyang Technological University 50 Nanyang Avenue Singapore 639798 Singapore; ^3^ Center for Advanced Mechanics and Materials Applied Mechanics Laboratory Department of Engineering Mechanics Tsinghua University Beijing 100084 China; ^4^ 2CSIJRI‐Qingyang Innovation Joint Lab China‐Singapore International Joint Research Institute Guangzhou Guangdong 510555 China

**Keywords:** alloy design, eutectic high‐entropy alloy, laser powder bed fusion, maximal strength, molecular dynamics

## Abstract

Eutectic alloys have driven technological advancements for centuries, from early bronze tools that marked the dawn of metallurgy to high‐performance soldering materials. Building on this legacy, eutectic high‐entropy alloys (EHEAs) have recently emerged to push the boundaries of mechanical performance. However, the strength potential of EHEAs remains largely untapped, primarily because of limitations in cooling rates, posing a significant challenge to the development of ultra‐strong bulk EHEAs. This study employs large‐scale molecular dynamics simulations to uncover key insights into the design of EHEAs with exceptional mechanical performance. Simulations reveal that the maximum tensile strength occurs at a critical interphase boundary spacing, an order of magnitude larger than that observed in conventional alloys. Below this spacing, the governing mechanism shifts from the Hall–Petch strengthening to dislocation multiplication–mediated softening. Guided by the simulation insights, a tensile strength of 1.8 GPa is achieved for laser powder bed fusion–fabricated EHEAs. This strength approaches the theoretical limit and outperforms other state‐of‐the‐art as‐printed high‐entropy alloys. This work not only establishes a viable pathway for designing ultra‐strong EHEAs but also provides a promising avenue for addressing the long‐standing challenge of developing high‐performance as‐printed materials for aerospace and other demanding applications.

## Graphical abstract

This study uses molecular dynamics simulations to guide the design of EHEAs with superior mechanical performance. The simulations reveal a peak tensile strength at a critical interphase boundary spacing. Below this spacing, the governing mechanism shifts from the Hall–Petch strengthening to dislocation multiplication–mediated softening.  Guided by these insights, LPBF‐fabricated EHEAs achieve a tensile strength of 1.8 GPa, approaching the theoretical limit and exceeding other as‐printed high‐entropy alloys.

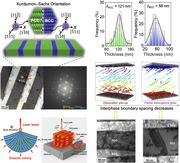

## 1 Introduction

Eutectic alloys have played a pivotal role throughout human history, from the cast irons that supported agricultural societies to the aluminum alloys used in modern manufacturing.^[^ ^1^, ^2^, ^3^, ^4^, ^5^ ^]^ Expanding upon this foundation, eutectic high‐entropy alloys (EHEAs) have emerged as cutting‐edge metallic materials. Characterized by multiple principal elements and unique phase composition, EHEAs have garnered significant attention for their outstanding mechanical properties.^[^ ^6^, ^7^, ^8^, ^9^ ^]^ A key factor in their performance lies in the interphase boundaries, where dislocation pile‐ups form, leading to interphase boundary strengthening.^[^ ^10^, ^11^, ^12^ ^]^ Despite this promising mechanism, its effectiveness at the nanoscale remains poorly understood. Moreover, the microstructural heterogeneity of EHEAs and the challenges associated with in situ microstructure observation complicate efforts to fully elucidate the role of interphase boundaries in governing deformation behavior.^[^ ^13^, ^14^ ^]^

The critical role of interphase boundaries in shaping mechanical performance has long been a focal point in the study of metallic materials. In traditional polycrystalline alloys, mechanical strength is predominantly governed by the interactions between dislocations and grain boundaries or other defects,^[^ ^15^, ^16^, ^17^ ^]^ resulting in the well‐known Hall–Petch strengthening effect. This mechanism generally leads to increased strength as the grain size decreases. However, when grain sizes are reduced to nanometer scale, the Hall–Petch effect can break down because of grain boundary–assisted deformation^[^ ^18^, ^19^, ^20^ ^]^ or dislocation nucleation.^[^ ^21^ ^]^ For nanolamellar materials such as EHEAs, the critical spacing at which maximal strength occurs remains elusive, primarily because of the limitations in cooling rates required for achieving refined microstructures during conventional solidification processes. Recent advancements in laser powder bed fusion (LPBF) additive manufacturing have significantly advanced microstructural refinement strategies by leveraging its ultrahigh cooling rates.^[^ ^22^, ^23^, ^24^, ^25^ ^]^ Unlocking the strength potential of as‐printed EHEAs while ensuring sufficient uniform tensile strain is crucial for meeting the demands of advanced applications.

In this study, we explored the strength potential of the AlCoCrFeNi_2.1_ EHEA system using large‐scale molecular dynamics (MD) simulations and LPBF additive manufacturing. The AlCoCrFeNi_2.1_ EHEAs offer a broad processing window for LPBF manufacturing.^[^ ^22^ ^]^ Our simulations revealed a peak in tensile strength at a critical interphase boundary spacing approaching 100 nm, an order of magnitude larger than that observed in conventional alloys. Below this critical spacing, a shift occurred from the traditional Hall–Petch strengthening mechanism to a dislocation multiplication–mediated softening mechanism, differing from previously reported deformation mechanisms in metallic materials. We experimentally validated this phenomenon by examining the tensile properties of LPBF‐printed AlCoCrFeNi_2.1_ EHEAs. Guided by simulation insights, the strength potential of the alloy was maximized around the critical spacing, achieving a maximal strength of 1.8 GPa superior to that of other state‐of‐the‐art as‐printed high‐entropy alloys (HEAs) while maintaining a sufficient uniform tensile elongation (>8%). These findings can advance our understanding of nanoscale effects on the strength of metallic materials and offer potential design strategies for the rational design of EHEAs and other hetero‐structured alloys with exceptional mechanical properties.

## 2 Results and Discussion

### 2.1 Maximal Strength in Simulated Eutectic High‐Entropy Alloys

**Figure 1:**
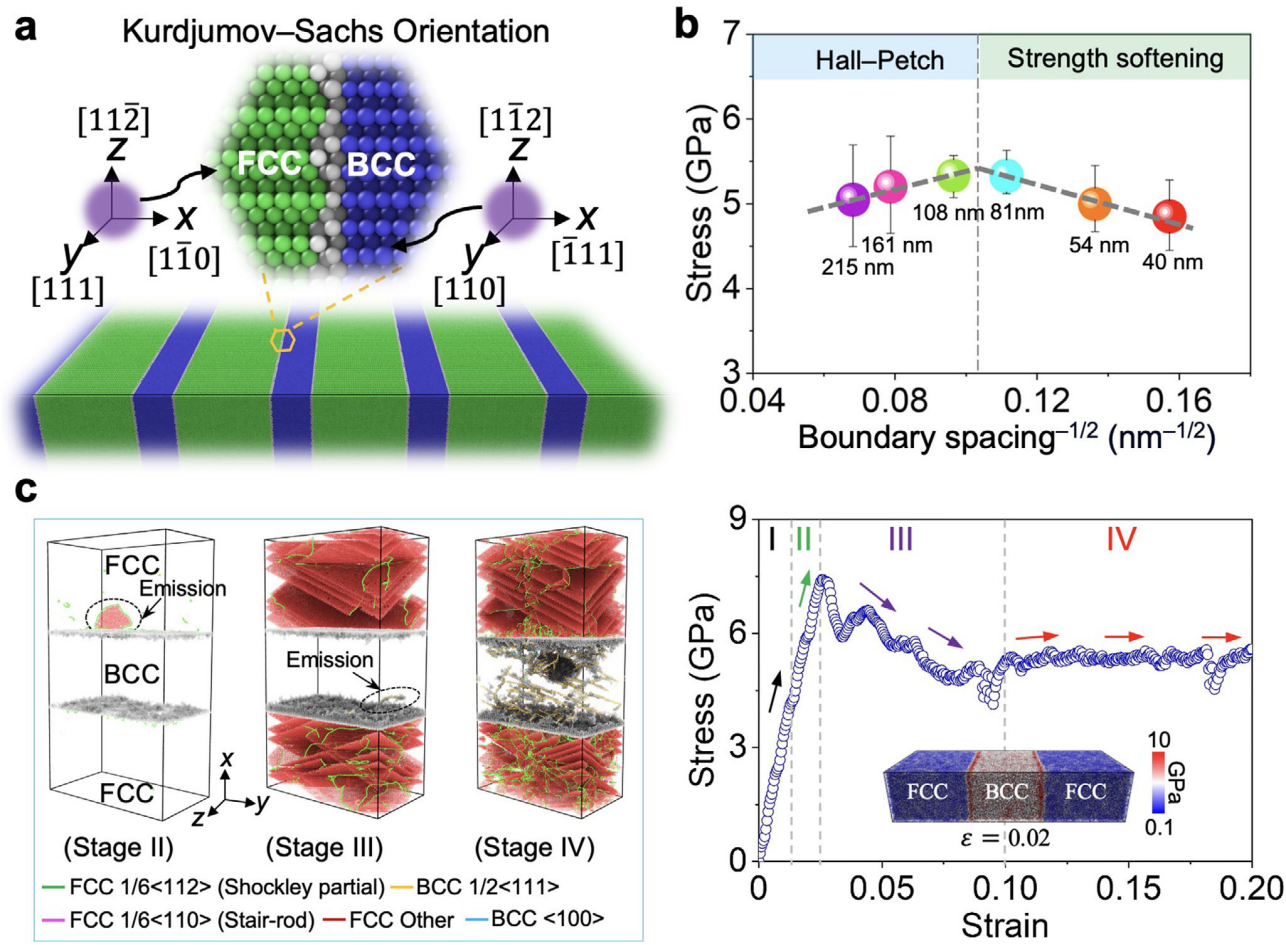
Atomistic simulations of uniaxial tensile deformation of AlCoCrFeNi_2.1_ EHEAs. a) A schematic atomic structure of the EHEAs with alternating FCC and BCC lamellae. The orientation of the FCC and BCC phases follows the Kurdjumov–Sachs relationship. b) Relationship between average flow stress with a strain range of 0.1–0.2 and interphase boundary spacing. c) Stress–strain response and deformation behavior of an EHEA with an interphase boundary spacing of 108 nm. The left panel shows the deformation behaviors at different stages. The stacking faults are marked by red atoms, and the dislocation lines are colored based on their slip planes. The right panel shows the stress–strain curve and von Mises stress distribution within the structure. The BCC phase contributes more significantly to the total von Mises stress.

So far, robust atomistic models for EHEA structures have not been established. To address this gap, we developed atomistic models that represent the interphase boundary structure of AlCoCrFeNi_2.1_ EHEAs (**Figure** **1**a). The semi‐coherent interface in the model is characterized by the Kurdjumov–Sachs relationship. We conducted uniaxial tensile deformation using MD simulations, and the results reveal a Hall–Petch relationship with increasing flow stress as the interphase boundary spacing decreases (Figure 1b and Figure S1, Supporting Information). This relationship persists until the interphase boundary spacing reaches a critical value, at which the maximal flow stress is found.

We investigated the progressive yielding and hardening behaviors of the face‐centered cubic (FCC) and body‐centered cubic (BCC) phases within the AlCoCrFeNi_2.1_ EHEA structure and compared the simulations against previous experiments. The stress–strain responses in Figure 1c reveal four distinct stages. In stage I, both the FCC and BCC phases undergo elastic deformation. In stage II, yielding occurs in the softer FCC phase, generating Shockley partial dislocations with a Burges vector of [1$\bar{1}$2]/6 from the phase boundaries. During this stage, yielding does not occur in the harder BCC phase, which imposes a geometric constraint on the softer FCC phase, leading to a local plastic strain gradient. Subsequently, geometrically necessary dislocations (GNDs) are formed in the FCC phase and piled up against the phase boundaries to accommodate the heterogeneous deformation, leading to strengthening. In stage III, yielding also occurred in the BCC phase, causing the emission of Shockley partial dislocations with a Burgers vector of [111]/2 from the phase boundaries. Deformation‐induced stacking faults (SFs) are formed in the FCC phase and become more prevalent with increasing strain. In stage IV, the heterogeneous deformation between the yielded FCC and BCC phases persists, promoting the accumulation of GNDs at the phase boundaries and resulting in strain hardening. These findings are consistent with the deformation mechanisms observed in previous experiments.^[^ ^16^, ^22^, ^26^ ^]^

### 2.2 Atomistic Insights into the Deformation Mechanisms

**Figure 2:**
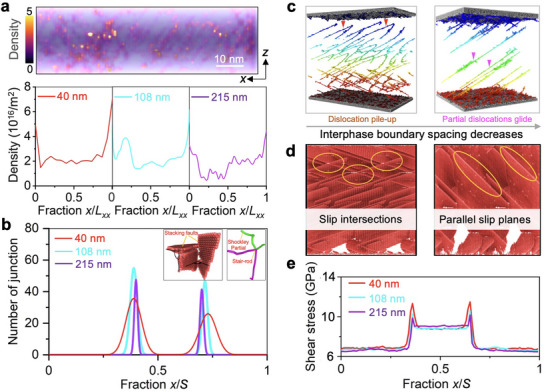
Atomistic insights into the deformation mechanisms. a) Distribution of dislocations at 10% strain. The top panel shows the density distribution of dislocations across an FCC lamella, while the bottom panel shows the curves of dislocation density plotted against the distance fraction *x*/*L*_FCC_, with *x* denoting the distance from the left boundary and *L_FCC_* the total length of FCC lamella. b) Distribution of junctions (stair‐rod dislocation) plotted against the distance fraction at 10% strain, where *S* is the total length of FCC and BCC lamellae. c) Snapshots of the transition in the dislocation mechanism from dislocation pile‐up (*S* = 108 nm) marked by red triangles to partial dislocation glide (*S* = 40 nm) marked by purple triangles. Colors are assigned to visualize the defects based on the spatial coordinates of atoms. d) Snapshots of the transition in deformation characteristics from slip intersections (*S* = 108 nm) to parallel slip planes (*S* = 40 nm). e) Distribution of von Mises stress at different interphase boundary spacings at 10% strain.

We investigated the origin of maximal strength by analyzing the evolution of GNDs in relation to the interphase boundary spacing. To accommodate the heterogeneous deformation within the EHEA structure, GNDs were found to nucleate and pile up against the interphase boundaries, leading to a high dislocation density near the boundaries (**Figure** **2**a). It is shown that there exists a critical spacing at which the density of GNDs reaches the maximum. Above this threshold, GND density decreases as the spacing decreases. This critical spacing aligns with the point where the maximal flow stress is observed. These increasing GNDs can counteract the applied shear stress and inhibit the movement of nearby dislocations, leading to enhanced strain hardening.^[^ ^27^ ^]^ Figure 2b shows the correlation between the nucleation of stair‐rod dislocations and interphase boundary spacing. As the spacing decreases in the Hall–Petch regime, there is an increase in the density of stair‐rod dislocations near the interphase boundaries, which could be ascribed to the increasing strain gradient near the boundaries due to the heterogenous deformation between the FCC and BCC phases.

When the maximal strength is achieved, a fundamental shift in dislocation behavior within the FCC lamellae starts to be activated (Figure 2c), transitioning from dislocation pile‐up to partial dislocations gliding along the interphase boundaries. Simulations show the multiplication and gliding of partial dislocations after plastic deformation, with these dislocations aligning on the same slip planes because of limited spacing (Figure 2d). During the dislocation glide, the interactions between the partial dislocations on parallel planes were reduced, as evidenced by the decrease in stair‐rod dislocation density in Figure 2b. Moreover, the stress field generated by the partial dislocations can locally shear the interphase boundaries (Figure 2e) and further promote the multiplication of partial dislocations from the boundaries, discouraging strain hardening.^[^ ^28^ ^]^

The maximal strength is closely linked to the characteristics of the interphase boundaries. In AlCoCrFeNi_2.1_ EHEAs, the interphase boundaries between the FCC and BCC phases form a semi‐coherent interface. Misfit dislocations are generated within these boundaries to relieve coherency strain at a certain distance away from the interphase. During deformation, these misfit dislocations act as potential sites for additional dislocation multiplication (Figure S2, Supporting Information). Because of limited spacing, the interphase boundaries impede the motion of dislocations toward them, forcing partial dislocations to glide along the boundaries. Additionally, the intermixing of elements within these boundaries, driven by the negative heat of mixing characteristic of high‐entropy materials, can reduce dislocation traps through core spreading and facilitate smoother dislocation glide.^[^ ^29^ ^]^

### 2.3 Tensile Properties of as‐Printed Eutectic High‐Entropy Alloys

**Figure 3:**
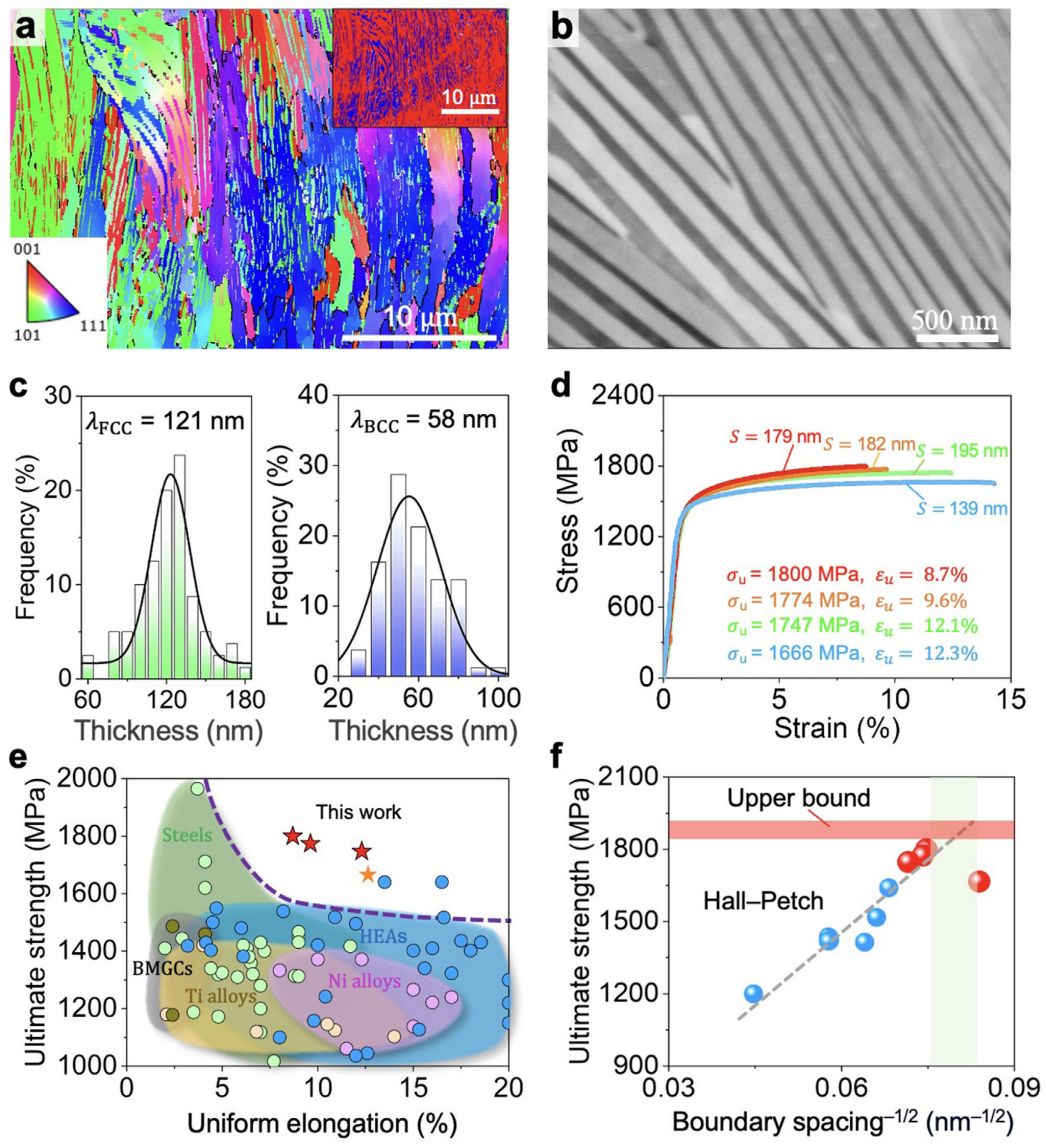
Microstructure and tensile properties of as‐printed AlCoCrFeNi_2.1_ EHEAs. a) EBSD inverse pole figure map of the cross‐section. The inset displays a two‐color EBSD map, with alternating FCC and BCC phases marked in red and blue, respectively. b) SEM–back‐scattered images of the alternating FCC (bright contrast) and BCC (dark contrast) lamellae with an average interphase boundary spacing *S* of ≈179 nm. c) Thickness distribution of the FCC and BCC lamellae. d) Tensile stress–strain curves with various *S*. The ultimate strength *σ*_u_ and uniform elongation *ε*_u_ are shown. e) Ultimate tensile strength versus uniform elongation in the Hall–Petch regime (red stars) and strength‐softening regime (orange star), and other high‐performance as‐printed alloys, including other HEAs, Ti alloys, Ni alloys, steels, and bulk metallic glass composites (BMGCs). f) Ultimate tensile strength versus interphase boundary spacing for reported as‐printed AlCoCrFeNi_2.1_ EHEAs (blue circles) and our specimens (red circles). The upper bound is established by fitting the tensile strength to the inverse square root of the interphase boundary spacing within the Hall–Petch regime.

With insights derived from the simulations, we adopted LPBF additive manufacturing to fabricate the AlCoCrFeNi_2.1_ EHEAs because of its ultrahigh cooling rates, allowing for refined lamellae spacing. By varying the laser power, scanning speed, and melting times for each layer, we were able to obtain different interphase boundary spacings within the as‐printed AlCoCrFeNi_2.1_ EHEAs. The electron backscatter diffraction (EBSD) inverse pole figure map in **Figure** **3**a shows that the as‐printed AlCoCrFeNi_2.1_ EHEA displayed eutectic colonies composed of dual‐phase lamellae. Because of the semicircular geometry of melt pools and the 67° rotation scanning strategy employed in the LPBF process, these eutectic colonies elongate toward the center of melt pools, with a random distribution of crystallographic orientation along the tensile direction, (Figure S3, Supporting Information), which can minimize anisotropic mechanical properties in the direction parallel to the tensile direction.^[^ ^22^ ^]^ Scanning electron microscope (SEM)–back‐scattered electron images confirmed a dual‐phase structure consisting of alternating FCC and BCC lamellae (Figure 3b,c) with nanoscale interphase boundary spacings (Figure S4, Supporting Information). It is shown that the unique feature of LPBF unlocks the possibility of investigating the mechanical behaviors of EHEAs at the nanoscale (i.e., when the lamella thickness is smaller than 100 nm), a previously inaccessible dimension due to the limitations of traditional solidification techniques.

The tensile stress–strain curves of the as‐printed AlCoCrFeNi_2.1_ EHEAs are shown in Figure 3d. Compared to other state‐of‐the‐art additively manufactured alloys, the EHEAs exhibit good strength–ductility synergy (Figure 3e and Table S1, Supporting Information). In agreement with the simulation results, a maximal tensile strength occurred around the critical interphase boundary spacing. Remarkably, Figure 3f demonstrates that the highest tensile strength achieved in this study closely approaches the theoretical upper bound for the as‐printed AlCoCrFeNi_2.1_ EHEA system, as estimated by the Hall–Petch relationship between tensile strength and the inverse square root of boundary spacing (Table S2, Supporting Information). Consequently, the smaller boundary spacing attained here, relative to previously reported results, effectively maximizes the strength potential of the eutectic system. Note that the Hall–Petch relationship has been extended to describe the full stress–strain behavior, considering that both boundary strengthening and dislocation strengthening scale proportionally with the inverse square root of grain size.^[^ ^30^ ^]^

In the Hall–Petch regime, our as‐printed AlCoCrFeNi_2.1_ EHEAs exhibit outstanding tensile strength without significantly compromised ductility, thus exhibiting good strength–ductility synergy for AlCoCrFeNi_2.1_ EHEAs compared to previous benchmark studies.^[^ ^22^ ^]^ Additionally, our results indicate that optimizing nanoscale lamellar thickness is effective to maintain the strength–ductility synergy without requiring post‐treatment. However, the observed strength softening led to a noticeable reduction in tensile strength, resulting in diminished strength–ductility synergy. This phenomenon can be attributed to reduced strain hardening associated with the decreased pile‐up of GNDs.

### 2.4 Microstructural Characterization

**Figure 4:**
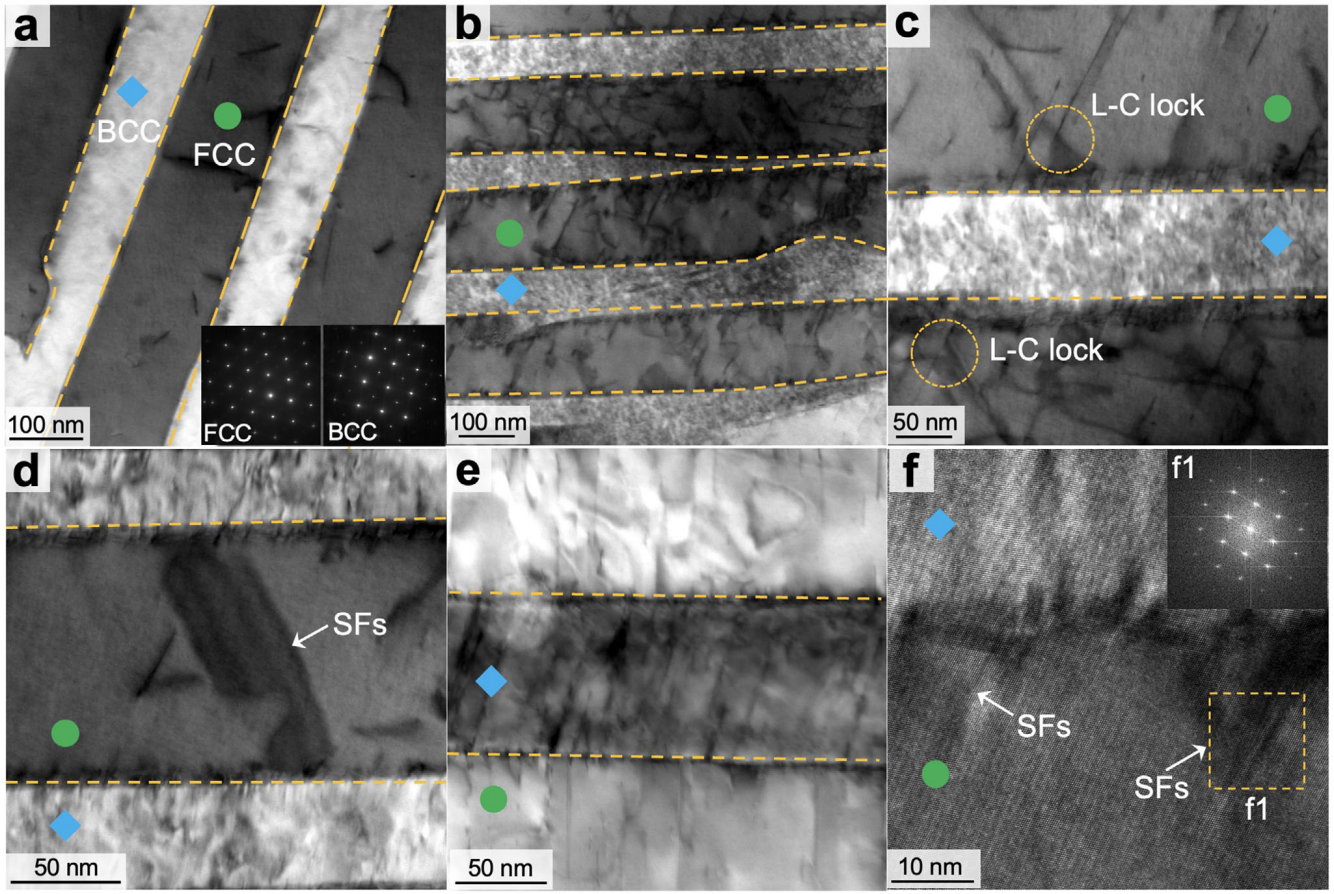
Bright‐field scanning transmission electron microscopy (STEM) images of as‐printed AlCoCrFeNi_2.1_ EHEAs. a) STEM image of the FCC and BCC lamellae with the inset illustrating the Kurdjumov–Sachs relationship at the interface. The FCC and BCC phases are marked by green and blue dots, respectively. b) Pronounced dislocation multiplications in the FCC lamellae. c) Formation of L–C locks through the interaction of SFs. d) Stacking faults connecting two adjacent phase boundaries in the FCC lamellae. e) Dislocations in the BCC lamellae. f) HRTEM image showing the FCC and BCC phase interface and the deformation‐induced stacking faults. The insets (f1) show the FFT micrographs of the SFs.

We used transmission electron microscope (TEM) to investigate the deformation mechanism of the AlCoCrFeNi_2.1_ EHEAs. The as‐printed samples exhibited the Kurdjumov–Sachs relationship between the FCC and BCC phases at their interface (**Figure** **4**a). In contrast to conventionally fabricated EHEAs where the BCC lamellae consist of an ordered B2 with disordered BCC nanoprecipitates,^[^ ^26^ ^]^ the EHEAs exhibit a disordered BCC matrix with ordered B2 nanoprecipitates (Figure S5, Supporting Information). In conventional fabrication methods, such as casting, elemental diffusion occurs relatively freely during solidification due to lower cooling rates. This allows sufficient time for the formation of equilibrium ordered B2 phase as the matrix, accompanied by the precipitation of disordered BCC phase. In contrast, the ultra‐high cooling rates characteristic of LPBF processes (10⁶–10⁸ K s^−1^) significantly suppress atomic diffusion. Under these conditions, rapid solidification can shift the phase formation mechanism toward diffusion‐limited solidification, drastically restricting elemental partitioning and chemical ordering.^[^ ^31^ ^]^ Consequently, the resulting matrix tends to form a disordered BCC matrix due to insufficient time for ordered‐phase formation.

When the interphase boundary spacing was within the Hall–Petch regime, the dislocations tended to accumulate near the phase boundaries in response to the local plastic strain gradient caused by the heterogeneous deformation (Figure 4b). Extensive dislocation multiplications were also observed within both the BCC and FCC lamellae after plastic deformation (Figure 4b,c). The dislocations within the BCC lamellae exhibited a wavy and isotropic network, which suggests an entanglement between edge and screw components. Such an isotropic dislocation network can maintain a more stable plastic flow within the BCC lamellae,^[^ ^32^, ^33^ ^]^ thus leading to enhanced strain hardening.

During plastic deformation, stair‐rod dislocations were formed (Figure 4c) through the interactions between SFs:

(1) $$\frac{a}{6}\left[2\bar{1}\bar{1}\right]+\frac{a}{6}\left[\bar{1}21\right]\to \frac{a}{6}\left[110\right]$$

where *a* is the lattice parameter. These dislocations exhibit a strong pinning effect, which can contribute to the formation of dislocation pile‐ups, resulting in strain hardening.^[^ ^34^, ^35^ ^]^ These findings corroborate the strengthening mechanism observed in the simulations (Figure 2b).

The maximal strength is induced by the transition from dislocation pile‐ups to dislocation multiplication in which multiple parallel partial dislocations/stacking faults were present and glided along the interphase boundaries during plastic deformation (Figure 4d). Consequently, the interactions between the partial dislocations reduced because of their alignment on parallel slip planes compared to the stair‐rod dislocations, leading to potential strength softening. In contrast to the isotropic dislocation network previously observed within the BCC lamellae, the dislocations exhibited a less isotropic configuration due to the geometrical confinement of the nanoscale lamellae (Figure 4e), which resulted in less stable plastic flow during deformation. The stress field generated by the partial dislocations can also locally shear the interphase boundaries, leading to a twisted interface (Figure 4f), in agreement with the simulation result (Figure S2, Supporting Information). The transition in the deformation mechanism from Hall–Petch strengthening to dislocation multiplication–mediated softening also provides strong support for the simulation results (Figure S6, Supporting Information).

The deformation mechanism observed here differs significantly from the conventional deformation mechanism observed in nanocrystalline materials, which is driven by grain boundary–assisted deformation,^[^ ^18^, ^19^, ^20^ ^]^ and in metallic multilayer materials, where slip transmission dominates.^[^ ^36^, ^37^ ^]^ It is distinct from the mechanism observed in nano‐twinned metals, although both phenomena bear resemblance in some aspects. In nano‐twinned metals, the maximal strength is driven by dislocation nucleation–induced twin‐boundary migration.^[^ ^21^ ^]^ In our study, the influence of dislocation nucleation is minimal, as reflected by the limited changes in yield strength (Figure 3d), indicating that other factors, such as dislocation multiplication along interphase boundaries, play a more critical role. It is acknowledged that variations in printing conditions may cause slight differences in phase fractions, lamellar length, regularity, and continuity among samples. Notwithstanding, our analyses suggest that these variations are not the dominant factors influencing the strength‐softening mechanism observed in our experiments (Figures S7–S9, Table S3, Supporting Information). For instance, while the decrease in FCC correlates well with the increase in ultimate tensile strength for the samples with a lamellae thickness above the critical distance, it fails to explain the strength‐softening mechanism revealed in this manuscript (Table S3, Supporting Information).

## 3 Discussion

### 3.1 Effect of the Critical Size on Maximal Strength

Traditional nanocrystalline or nano‐twinned alloys can exhibit maximal strength when the grain sizes or twin thickness falls below a few tens of nanometers, a principle that has long guided the design of high‐strength materials at the nanoscale. In contrast, our work reveals that the critical size for achieving the maximal strength in EHEAs can be an order of magnitude larger than that observed in conventional materials.

Such critical size is associated with the SF energy (SFE) of the EHEAs. It has been proposed that there is a critical spacing for metallic multilayer materials below which multiplication and movement of Shockley partial dislocations prevail over full dislocations.^[^ ^38^ ^]^ According to the classic dislocation theory, the critical thickness for the FCC lamellae in the EHEAs can be estimated as ^[^ ^39^ ^]^

(2) $$h=\frac{2\alpha \mu \left(b_{f}-b_{p}\right)b_{p}}{\gamma }$$

where *μ* is the shear modulus (81 GPa), *b_f_* (0.254 nm), and *b_p_* (0.147 nm) are the magnitudes of a full dislocation and Shockley partial dislocation, respectively, and *γ* is the SFE of the FCC phase (≈22 mJ m^−2^ from our density functional theory calculation). The parameter α reflects the dislocation characters (*α* = 0.5 and 1.5 for edge and screw dislocations, respectively). Typically, the twinning‐induced plasticity effect will be activated when the SFE is below ≈21 mJ m^−^^2^, and the transformation‐induced plasticity effect will be activated at even lower SFEs (below ≈8 mJ m^−^^2^).^[^ ^40^ ^]^ The calculated SFE in the FCC phase of AlCoCrFeNi_2.1_ EHEA is consistent with the formation of SFs in our TEM observations. Here, we adopt *α* = 1 for the general situation. The critical interphase boundary spacing for the multiplication of Shockley partial dislocations is calculated as ≈166 nm, which aligns well with our experimental results. Accordingly, the critical feature size for the maximal strength can be calculated for well‐known metallic materials, such as ≈7 nm for Al/Ti lamellar alloys (SFE = 253 mJ m^−2^) and ≈18 nm for nano‐twinned Cu (SFE = 80 mJ m^−2^), both of which align with previous experimental findings.^[^ ^21^, ^38^ ^]^ Compared to the Al/Ti lamellar alloys and nano‐twinned Cu, the relatively lower SFE of the FCC phase in the AlCoCrFeNi_2.1_ EHEAs plays an important role in the larger critical size of the maximum strength.

The calculated critical interphase boundary spacing from simulations deviates from the experimental findings, which may be attributed to defects formed during the LPBF process. Such defects can influence the accumulation of GNDs, consequently altering the experimentally determined optimal interphase boundary spacing. Additionally, inherent limitations of MD simulations, such as idealized atomic configurations and limited spatial scale, may further contribute to the observed discrepancy. Nevertheless, it is generally believed that MD simulations remain valuable for qualitatively capturing deformation mechanisms and elucidating the relationship between interphase boundary spacing and mechanical strength.

### 3.2 Design Strategies Based on the Hetero‐Boundary‐Affected Zone

For EHEAs, the interphase boundary wields a profound influence over the mechanical performance. Because of discontinuous slip planes, the semi‐coherent interphase boundary commonly requires a high critical stress for slip transmission. Consequently, the pile‐up of GNDs against the interphase boundary can generate a long‐range hetero‐deformation induced (HDI) stress, leading to stress partitioning and strain hardening.^[^ ^41^, ^42^ ^]^ When the interphase boundary spacing is sufficiently large, the generation of HDI stress within the lamellae will be confined to the affected zone, rendering other regions ineffective in facilitating HDI strain hardening. Such an affected zone can be defined as the region exhibiting a strain gradient near the boundaries, also known as the hetero‐boundary‐affected zone in hetero‐structured materials.^[^ ^41^ ^]^

Based on the dislocation pile‐up theory, we propose the existence of an optimal hetero‐boundary‐affected zone within the EHEAs that exhibits the highest strain gradient near the boundaries with a fixed relative thickness ratio between the soft FCC phase and the hard BCC phase. When the interphase boundary spacing is smaller than this zone, the strain gradient will diminish because of the glide of Shockley partial dislocations along the interphase boundaries instead of dislocation pile‐up. The relative thickness ratio can also play a crucial role in mechanical properties, as it directly affects both dislocation pile‐up and dislocation nucleation.^[^ ^43^ ^]^ However, achieving precise control over this ratio remains challenging in LPBF because of the inherently rapid solidification dynamics. Further research is warranted to optimize this ratio and the mechanical performance of EHEAs.

**Figure 5:**
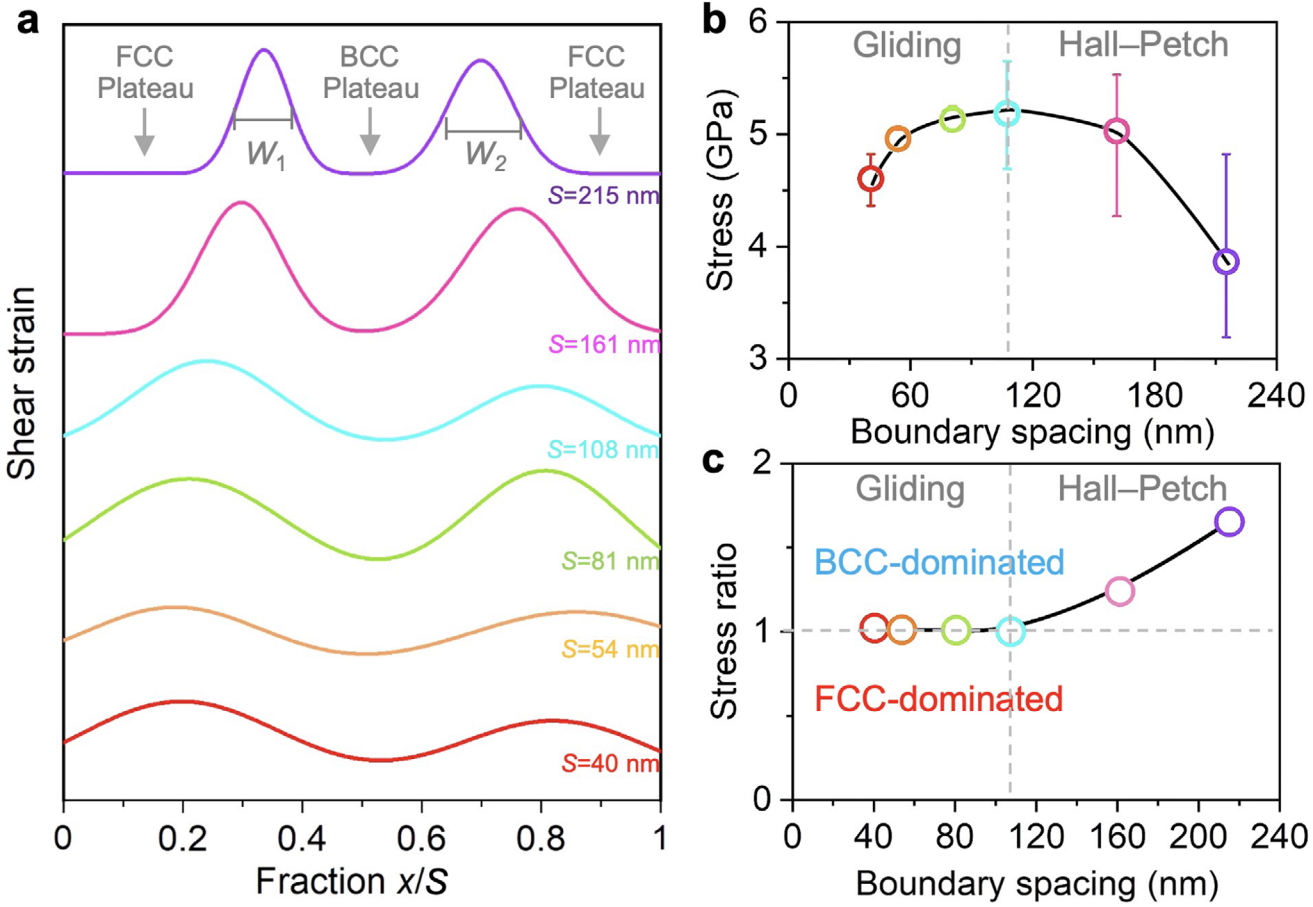
Hetero‐boundary‐affected zone and its relationship with the onset of dislocation multiplication–mediated strength softening. a) Distribution of von Mises shear strain versus distance fraction *x*/*S* for different interphase boundary spacing *S*. *W*_1_ and *W*_2_ are the full width at half maximum. b) Tensile stress within the FCC lamellae as a function of the interphase boundary spacing at 15% strain. c) Ratio between the tensile stress within the BCC and FCC phases at 15% strain. The BCC phase contributes more significantly to the overall tensile stress in the Hall–Petch regime.

We calculate the width of the hetero‐boundary‐affected zone obtained from the MD simulation results by fitting the strain peaks across the boundaries using a Gaussian distribution function (**Figure** **5**a). In this context, *H* represents the intensity of the strain peak at the interface, and *W* denotes the full width at half maximum (*H*/2), which can be considered as the proportion of the hetero‐boundary‐affected zone to the FCC lamellae. In the Hall–Petch regime, it is shown that the proportion of the hetero‐boundary‐affected zone grew as the interphase boundary spacing decreased, leading to an increased capacity of FCC lamellae to generate internal stress (Figure 5b). This finding corroborates the observed increase in the density of GNDs as the interphase boundary spacing decreased (Figure 2a). As the proportion of the hetero‐boundary‐affected zone expands, the density increases, providing more potential locations for the pile‐up of GNDs.^[^ ^41^ ^]^

Based on the dislocation pile‐up theory, the critical width of hetero‐boundary‐affected zone *L* can be estimated as ^[^ ^42^ ^]^

(3) $$L=\frac{\mu nb}{\pi \left(1-\nu \right)\sigma }$$

where *μ* is the shear modulus, *n* is the average number of dislocation pile‐ups within the lamellae, *b* is the length of the Burgers vector, *ν* is the Poisson ratio, and *σ* is the applied stress. According to previous experimental measurements,^[^ ^22^, ^44^ ^]^ the value of *L* is approximated as 33 nm. The interphase boundary spacing is the combined length of one FCC and one BCC lamella with a constant FCC over BCC ratio of 2.36. The corresponding interphase boundary spacing is calculated as *S* = 2*L/ζ* = 94 nm, where *ζ* is the ratio of the FCC lamellae thickness to the interphase boundary spacing. This is consistent with our simulation results (Figure 1b). In the Hall–Petch regime, it was observed that stress partitioning between FCC and BCC lamellae decreased as the interphase boundary spacing was reduced (Figure 5c). Under these conditions, the lamellae would gradually lose their capability to efficiently distribute strain among distinct microstructural regions. Consequently, deformation would become localized, potentially leading to premature failure and diminished ductility, as confirmed by the tensile properties presented in Figure 3d. When the interphase boundary spacing fell below the critical width of the hetero‐boundary‐affected zone, internal stress partitioning began to homogenize because of the activation of dislocation multiplication mechanisms. This phenomenon would suppress strain hardening, ultimately diminishing the strength–ductility synergy, as clearly illustrated in Figure 3d.

In conclusion, we have demonstrated the strength potential of the AlCoCrFeNi_2.1_ EHEAs through a combination of large‐scale MD simulations and LPBF additive manufacturing. Simulations reveal that the tensile strength is maximized at a critical interphase boundary spacing due to the transition from Hall–Petch strengthening to dislocation multiplication–induced softening. Such critical size is an order of magnitude larger than that observed in conventional nanocrystalline and nano‐twinned materials. This phenomenon can be attributed to the relatively low SFE of the EHEAs compared to other conventional alloys, which allows for a larger critical size that favors the Shockley partial dislocations over full dislocations within the nanolamellae. These findings are consistent with our experimental results. Leveraged from the simulation insights, the LPBF‐fabricated AlCoCrFeNi_2.1_ EHEA system approaches its theoretical strength limit of 1.8 GPa around the critical interphase boundary spacing, outperforming other state‐of‐the‐art as‐printed high‐entropy alloys. This study not only broadens our understanding of nanoscale effects on material strength but also provides a promising avenue for addressing the long‐standing challenge of developing ultra‐high strength metallic materials in as‐printed condition with sufficient uniform tensile strain. The design strategies proposed in this work can pave the way for the rational design of high‐strength EHEAs and other similar hetero‐structured materials in potential industrial applications.

## 4 Experimental Section

### Materials Fabrication

Gas‐atomized AlCoCrFeNi_2.1_ EHEA powders with a size range of 20–53 µm were employed to produce samples for microstructure characterization and mechanical tests. The nominal composition of the powder is shown in Table S4 (Supporting Information). The LPBF machine operated was EOS M290 equipped with a 400‐W ytterbium‐fiber laser with a wavelength of 1060 nm. The laser beam was focused at a size of 70 µm. All the printing was conducted in an argon atmosphere. The steel baseplate was pre‐heated to 80 °C prior to the printing process. All the samples were printed using a constant laser power of 350 W, layer thickness of 40 µm, and a rotation of 67° (Table S5, Supporting Information). To vary the thickness of the lamellar structure in the EHEA samples, the laser scanning speed was changed between 600 and 1000 mm s^−1^. To further refine the lamellar thickness, a remelting strategy, which indicates the laser scanned each print layer twice, was also adopted on the print using a scanning speed of 1000 mm s^−1^. The first batch of cubic samples (10 × 10 × 10 mm^3^) was produced for checking the print quality and characterizing the microstructure. All samples produced by these process parameters had a relative density higher than 99.5% (Table S6, Supporting Information). The geometry of the printed samples used for tensile test were 40 (length) × 10 (width) × 10 (height) mm^3^.

### Mechanical Testing

The dog‐bone shaped tensile specimens with a gauge dimension of 8 mm (length) × 2 mm (width) × 1 mm (thickness) were cut from the printed samples by wire‐electrical discharge machining and finally polished using a 1200‐grit silicon carbide paper. Each type of sample was tested three times. Quasi‐static uniaxial tension tests were performed on a SHIMADZU AG‐X plus machine with a TRViewX video extensometer. The tensile strain rate was set at 1 × 10^−3^ s^−1^.

### Microstructure Characterization

The microstructure of EHEA samples was characterized by scanning electron microscopy and electron backscatter diffraction (EBSD) techniques on a JOEL 7600 filed emission microscope. The step size used for EBSD was set at 0.06 µm. The raw EBSD data was analyzed by the software Channel 5 (Oxford Instruments). Dislocation behavior was characterized using a FEI Talos F200 transmission electron microscope (TEM). Disk‐shaped TEM specimens, ≈3 mm in diameter, were prepared using a Twin‐jet electropolisher (Tenupol‐5, Denmark).

### Molecular Dynamics Simulation

The chemical composition and the ratio of lamella thickness between the FCC and BCC phases were adopted based on previous experimental measurements (Table S7, Supporting Information).^[^ ^22^ ^]^ The molecular dynamics simulations employed empirical embedded atom method (EAM) potentials to model the mechanical behavior of AlCoCrFeNi_2.1_ EHEAs.^[^ ^45^, ^46^, ^47^, ^48^ ^]^ To ensure reliable results, the potentials were carefully validated through a combination of experimental results and density functional theory calculations (Figure S10, Tables S8–S14, Supporting Information). In the large‐scale simulations, the EHEA structures are initially free of dislocations because the strength contribution from pre‐existing dislocations in the as‐printed specimen is much smaller than that from the deformation‐induced dislocations. A eutectic lamellar structure of AlCoCrFeNi_2.1_ was constructed by integrating an FCC lamella with a BCC lamella. The orientation relationship between the FCC and BCC phases was determined based on experimental findings,^[^ ^22^ ^]^ which followed the Kurdjumov–Sachs orientation relationship. The crystal orientation of the FCC phase was [1$\bar{1}$0], [111], and [11$\bar{2}$] along the *x*, *y*, and *z* directions, respectively, while the crystal orientation of the BCC phase was [$\bar{1}$11], [110], and [11$\bar{2}$] along the *x*, *y*, and *z* directions, respectively. Periodic boundary conditions were applied in all directions. Because of the spatial and temporal scale limitations inherent in MD simulations, modeling multiple lamellae with varying orientations remains computationally prohibitive. The simulations focus on the lamellar orientation perpendicular to the loading direction, which corresponds to the primary growth direction of the lamellae, as determined by the microstructural characterization (Figure S3, Supporting Information). In this orientation, stress partitioning between constituent phases is maximized, and dislocation pile‐up behavior is distinctly observable, offering valuable insights into the underlying deformation mechanisms. The EHEA structure has dimensions of 310 Å and 182 Å along the *y* and *z* directions, respectively. By varying the length in the *x* direction (interphase boundary spacing), various EHEA structures were obtained. The chemical composition and the ratio of lamella thickness between the FCC phase and BCC phase were adopted based on previous experimental measurements.^[^ ^22^ ^]^ The interphase boundary spacing is the combined length of one FCC and one BCC lamella with a constant FCC over BCC ratio of 2.36. The number of atoms in the eutectic system ranged from 1.5 to 20 million. The interphase boundary spacing was in the range of 40–215 nm, which encompassed the observed range in previous experimental work.^[^ ^22^ ^]^ The systems were energy‐minimized by performing the conjugate gradient algorithm and equilibrated at 300 K for 100 ps within the isothermal–isobaric (NPT) ensemble, i.e., constant number of atoms, pressure, and temperature. After the system was thermally equilibrated, uniaxial tensile simulation along the *x* direction was performed at a constant engineering strain rate of 5 × 10^8^ s^−1^, and the temperature was set at 300 K.^[^ ^49^ ^]^ It is noteworthy that it is computationally challenging for MD simulations to model a statistically representative three‐dimensional ensemble of free‐oriented lamellae at the length‐ and time‐scales relevant to the study. To assess the orientation sensitivity within these constraints, two bounding configurations were chosen: Lamellae boundary perpendicular to the loading axis, this minimizes the resolved shear stress along the interphase boundary; Lamellae boundary parallel to the loading axis, this maximizes the resolved shear stress along the interphase boundary. These two orientations bracket the full range of shear stress states that any arbitrary lamella could experience in uniaxial tension. Both simulations reveal the same softening mechanism (Figure S11, Supporting Information), indicating that the mechanism is applicable to the eutectic lamellae investigated in this work. Additionally, since the nanoprecipitates were identified in the experiment (Figure S5, Supporting Information), the influence of precipitates on the strength‐softening mechanism identified through MD simulations was also investigated. Combined simulation and experimental results indicate that precipitates do not play a governing role in the mechanism studied because of their limited quantity (Figure S12, Supporting Information). The open‐source software Large‐scale Atomic/Molecular Massively Parallel Simulator (LAMMPS)^[^ ^50^ ^]^ was used to carry out the simulations, and the Open Visualization Tool (OVITO) software ^[^ ^51^ ^]^ was used to visualize the atomic configurations and analyze the simulation results.

### Density Functional Theory Calculation

The Vienna ab initio simulation package (VASP)^[^ ^52^ ^]^ with a plane‐wave basis and projector augmented wave (PAW) potentials was used in this study. The generalized gradient approximation (GGA) in the form of the Perdew‐Burke‐Ernzerhof (PBE) functional for the exchange‐correlation energy potential was used.^[^ ^53^ ^]^ The bulk energy of the alloy system was obtained after geometry optimization with an energy convergence tolerance of 10^−8^ eV and a force convergence tolerance of 10^−4^ eV Å^−1^. A plane‐wave energy cutoff of 600 eV and k point mesh of 15 × 15 × 15 per cell were applied for the bulk system. The atomic structure was created using the supercell random approximates (SCRAPs) approach.^[^ ^54^ ^]^ The SFE was calculated using a FCC unit cell with 108 atoms in total with 9 layers along [111] axis and 12 atoms on each layer. A vacuum spacing of 15 Å along the [111] direction was added to avoid periodicity.

## Conflict of Interest

The authors declare no conflict of interest.

## Author Contributions

W.J., H.G., and K.Z. conceived the ideas and supervised the project. W.J., S.G., A.J., and X.S. prepared the alloys and performed the mechanical property tests. W.J. and A.J. conducted the simulation. W.J., S.G., A.J., H.G., Y.T., M.W., and K.Z. led the writing of the paper and the presentations in the figures. S.G. and X.S. performed TEM, STEM, and HRTEM experiments on dislocation configurations. All authors contributed to the discussion of the results and commented on the manuscript.

## Supporting information



Supporting Information

## Data Availability

The data that support the findings of this study are available from the corresponding author upon reasonable request.
