# Supplementary material for: Designing Maximal Strength in Nanolamellar Eutectic High‐Entropy Alloys
Source: Adv Mater. 2025 Jun 27;37(35):2500149. doi: 10.1002/adma.202500149 (PMC12411999; doi:10.1002/adma.202500149)
Supplement: Supplementary file 1 — Supporting Information [file ADMA-37-2500149-s001.docx]

**Supporting Information for**

**Designing Maximal Strength in Nanolamellar Eutectic High-entropy Alloys**

*Weiming Ji, Shubo Gao, Asker Jarlöv, Xiaojun Shen, Yujia Tian, Mao See Wu, Huajian Gao, Kun Zhou**

**Corresponding author. Email:* [*kzhou@ntu.edu.sg*](mailto:kzhou@ntu.edu.sg) *(K. Zhou)*

**1. Validation of the Atomic Potentials**

We have confirmed the reliability of the atomic potentials used in the study with density functional theory (DFT) calculations and experimental results from four different aspects: (1) lattice constants, (2) cohesive energy, (3) melting point, and (4) elastic constants. A good agreement in the lattice constants, cohesive energies, and melting points were obtained using the atomic potentials, demonstrating their reliability. The methodology is described as follows.

**1.1 Lattice constants**

The lattice constants of the AlCoCrFeNi_2.1_ eutectic high-entropy alloy (EHEA) structure after equilibration at 300 K were calculated and compared with the reported experimental results. The lattice mismatch was calculated according to


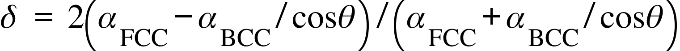
, (1)

where *α*_FCC_ and *α*_BCC_ are the lattice parameters of the face-centered cubic (FCC) and body-centered cubic (BCC) phases, respectively, and *θ* is the angle between the {111} and {110} planes. Supplementary Table S9 shows that the calculated lattice constants from molecular dynamics (MD) simulations coincide with the calculated DFT results.

**1.2 Cohesive energy**

DFT calculations were conducted to calculate the cohesive energy of all elemental pairs in binary L1_2_ AB_3_ and B2 AB structures for five elements (*i.e*., Al, Co, Cr, Fe, and Ni), using the Vienna *ab initio* simulation package (VASP) with a plane-wave basis and projector augmented wave (PAW) potentials. The generalized gradient approximation (GGA) in the form of the Perdew–Burke–Ernzerhof (PBE) functional for the exchange-correlation energy potential was used. For the binary-alloy L1_2_ AB_3_ system, the cohesive energy is calculated as


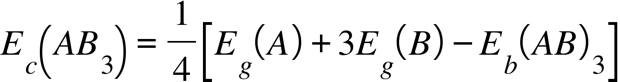
, (2)

where *E*_b_(AB)_3_ is the energy of the fully-relaxed L1_2_ AB_3_ structure, and *E*_g_(A) and *E*_g_(B) are the energy of isolated A and B atoms in their ground states, respectively. For the B2 AB system, the cohesive energy is calculated as


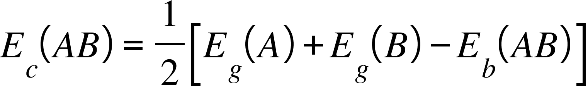
, (3)

where *E*_c_(AB) is the energy of the fully relaxed B2 AB structure.

To determine the energy of isolated A and B atoms in their ground state, DFT calculations were performed for a single atom in a large cubic cell with dimensions of 14 × 14 × 14 Å^3^. Periodic boundary conditions were applied to the simulation. Relaxation was achieved through the implementation of spin-polarized calculation with an energy convergence tolerance of 10^−8^ eV and a plane-wave energy cutoff of 800 eV for the single-atom system. The bulk energy of the alloy system was obtained after geometry optimization with an energy convergence tolerance of 10^−8^ eV and a force convergence tolerance of 10^−4^ eV Å^−1^. A plane-wave energy cutoff of 600 eV and k point mesh of 15 × 15 × 15 per cell were applied for the bulk system. Supplementary Tables S10−S13 show that the calculated binding energy from MD simulations coincides with the calculated results from DFT.

**1.3** **Elastic constants**

The elastic constants describe how much a material deforms when subjected to an external force. The elastic tensor of the EHEA was calculated at 0 K to avoid statistical fluctuations using the script implemented in the Large-scale Atomic/Molecular Massively Parallel Simulator (LAMMPS) software. The structure was first relaxed by geometry optimization with an energy tolerance and force convergence of 10^−10^ eV and 10^−10^ eV/Å, respectively. After relaxation, the elastic constants were calculated by measuring the change in the stress tensor when the structure was deformed in one direction. In addition, the bulk modulus of the eutectic structure is calculated. Table S14 shows that the calculated elastic constants coincide with the experimentally measured results.

**1.4 Melting point**

An EHEA structure was built with a unit cell of 133 × 108 × 119 Å^3^, and oriented with its *x*-, *y*- and *z*-directions along the [1$\bar{1}$0], [111], and [11$\bar{2}$] direction, respectively. Periodic boundary conditions were applied to all three directions. The FCC and BCC phases had a lamella thickness of 93 and 39.4 Å, respectively, leading to an FCC to BCC ratio of 2.36, consistent with previous experiments. The eutectic structure was equilibrated at 300 K for 100 ps, then heated to 1800 K in 1200 ps in the canonical (NVT) ensemble, *i.e.*, constant number of atoms, volume, and temperature, with a time step of 1 fs. During the heating process, the potential energy of the eutectic system was recorded. The dramatic change in potential energy signified the melting of the alloy, at which the melting temperature was obtained. Supplementary Figure S10 shows that the melting temperature is around 1568 K, which coincides with the experimentally measured temperature of 1616 K.

**2. Yield strength**

In our study, the change in yield strength could be explained by the change in lamellae thickness according to the Hall–Petch relationship.^[2,3]^ In general, the local stress acting on the leading dislocation in a pile-up is the applied shear stress $\tau$ magnified by a factor of $n$, which represents the number of pile-up dislocations. Plastic yielding occurs when this local stress reaches a critical value, leading to slip transmission across the phase interface. As a result, these shear stresses are related to the Hall–Petch equation according to^[2]^

$\tau=k_{0}S^{-1/2}=\frac{nGb}{4L}$, (4)

where $k_{0}$ is the strengthening coefficient, $L$ is the pile-up length and can be taken as half of the average thickness of FCC lamellae, $n$ is the average number of pile-up dislocations, $G$ is the shear modulus (81 GPa), and $b$ is the Burgers vector of the FCC phase (0.254 nm). Using the Taylor factor of the FCC phase (3.09),^[2]^ we can estimate the lamellar boundary strengthening.

The high density of pre-existing dislocations in the FCC lamellae makes an important contribution to the high yield strength of the as-printed EHEA. We estimated this strengthening effect using the Taylor hardening law and the rule of mixture for each phase^[2]^:

$\sigma_{\rho}=M\alpha Gb\rho^{1/2}$, (5)

where $M$ is the Taylor factor (3.09), $\alpha$ is the dimensionless pre-factor ($\alpha$ = 0.2 for FCC phase and 0.24 for BCC phase^[2]^), $b$ of FCC and BCC are 0.254 and 0.248 nm, respectively, $\rho$ is the pre-existing dislocation density. We can estimate the dislocation strengthening.

Additionally, the nanoprecipitate can also contribute to the high yield strength of the as-printed EHEA. Since the amount of L1_2_ nanoprecipitate is too small to be quantified, we mainly assess the amount of B2 nanoprecipitate based on statistical analysis of EDS mapping results (~14% for sample A). The yield strength increment caused by the coherency hardening $\sigma_{\mathrm{CS}}$, modulus hardening $\sigma_{\mathrm{MS}}$, and atomic order hardening $\sigma_{\mathrm{OS}}$ is given as^[1]^:

$\sigma_{\mathrm{CS}}=M\alpha_{\varepsilon}\left( G\varepsilon_{c} \right)^{\frac{3}{2}}{(\frac{rf}{0.5Gb})}^{\frac{1}{2}}$, (6)

$\sigma_{\mathrm{MS}}=0.0055M\left( \Delta G \right)^{\frac{3}{2}}{(\frac{2f}{G})}^{\frac{1}{2}}{(\frac{r}{b})}^{\frac{3m}{2}-1}$, (7)

$\sigma_{\mathrm{OS}}=0.81M\frac{\gamma_{APB}}{2b}{(\frac{3\pi f}{8})}^{\frac{1}{2}}$, (8)

where $\alpha_{\varepsilon}$ is a constant (2.6), $\varepsilon_{c}$ is the constrained lattice parameter mismatch (0.001), *r* is the mean radius of the precipitates (0.5 nm), *f* is the volume fraction of precipitate in its matrix (~0.14), $\Delta G$ is the shear modulus mismatch between B2 precipitate and its matrix (14 GPa ^[1]^), *m* is a constant (0.85), $\gamma_{APB}$ is the anti-phase boundary energy (198 mJ/m^2[11]^). The total strength increment from shearing of the precipitates is the larger one between $\sigma_{\mathrm{CS}}$ + $\sigma_{\mathrm{MS}}$ and $\sigma_{\mathrm{OS}}$. Based on the calculations, the $\sigma_{\mathrm{OS}}$ is the operative mechanism for B2 precipitates. Finally, the enhancement of strength $\sigma_{S}$ resulted from the nanoprecipitates can be estimated by the rule of mixture^[1]^:

$\sigma_{S}=f_{BCC}\sigma_{\mathrm{OS}}$, (9)

where $f_{BCC}$ is the phase fraction of BCC in the EHEA (~0.33 for sample A). The calculated $\sigma_{S}$ is around 120 MPa. With increasing cooling rates, atomic diffusion becomes progressively suppressed, limiting the formation of precipitates. Sample A, having the largest thickness and thus the lowest relative cooling rate, exhibits a maximum $\sigma_{S}$ of approximately 120 MPa, representative of all tested samples. Although minor variations in the quantity of nanoprecipitates may exist among the as-printed samples, their contribution to yield strength remains minimal compared to the dominant effects of lamellar boundaries and pre-existing dislocations. Therefore, we have adopted the calculated $\sigma_{S}$ as a representative value for all as-printed samples.

Finally, the yield strength of an alloy can be approximated according to

$\sigma_{y}=\sigma_{0}+\sigma_{\mathrm{LB}}+\sigma_{\rho}+\sigma_{S}$, (10)

where $\sigma_{0}$ is the friction stress ($\sigma_{0}$= 95 MPa ^[12]^). The results in Table S17 demonstrate a slight change in yield strength while still following the Hall–Petch relationship.

| 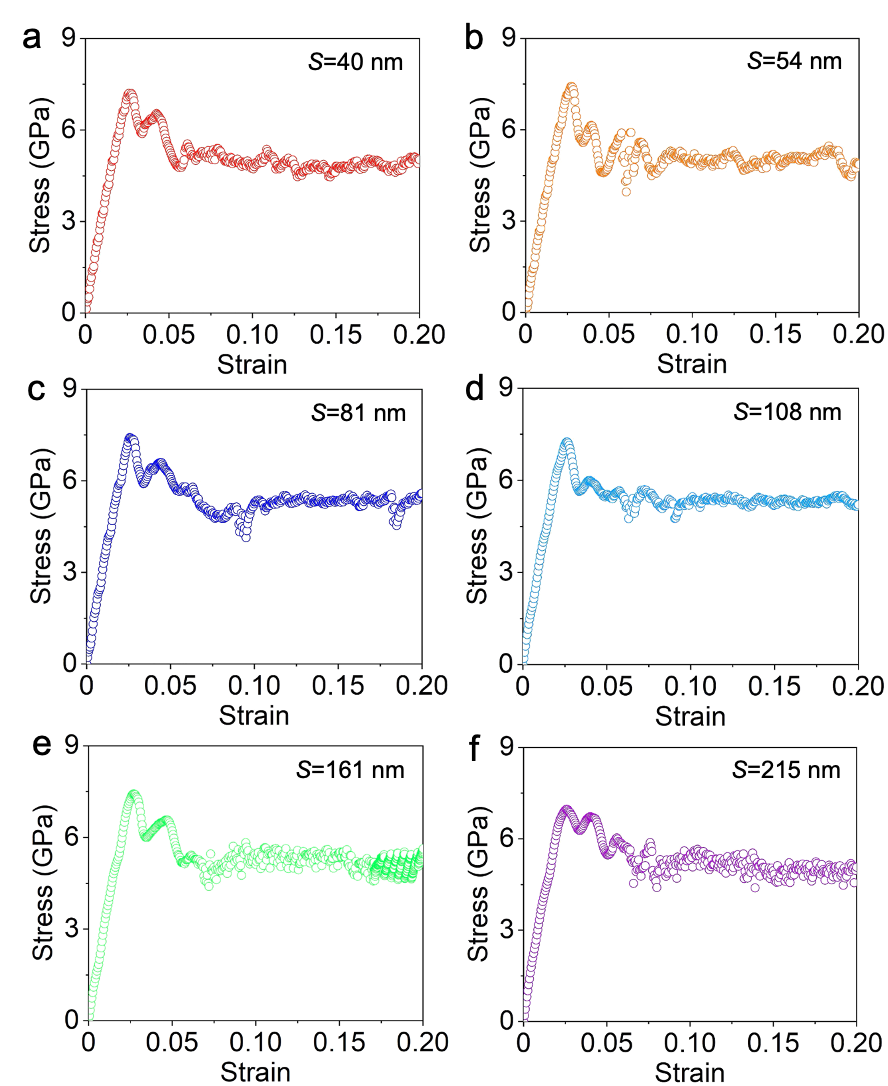 |
| --- |
| **Figure S1.** Simulated tensile stress–strain curves of the EHEAs as a function of interphase boundary spacing *S*. The stable flow stress over a strain range of 0.1–0.2 was used to calculate the average flow stress. |
| \| 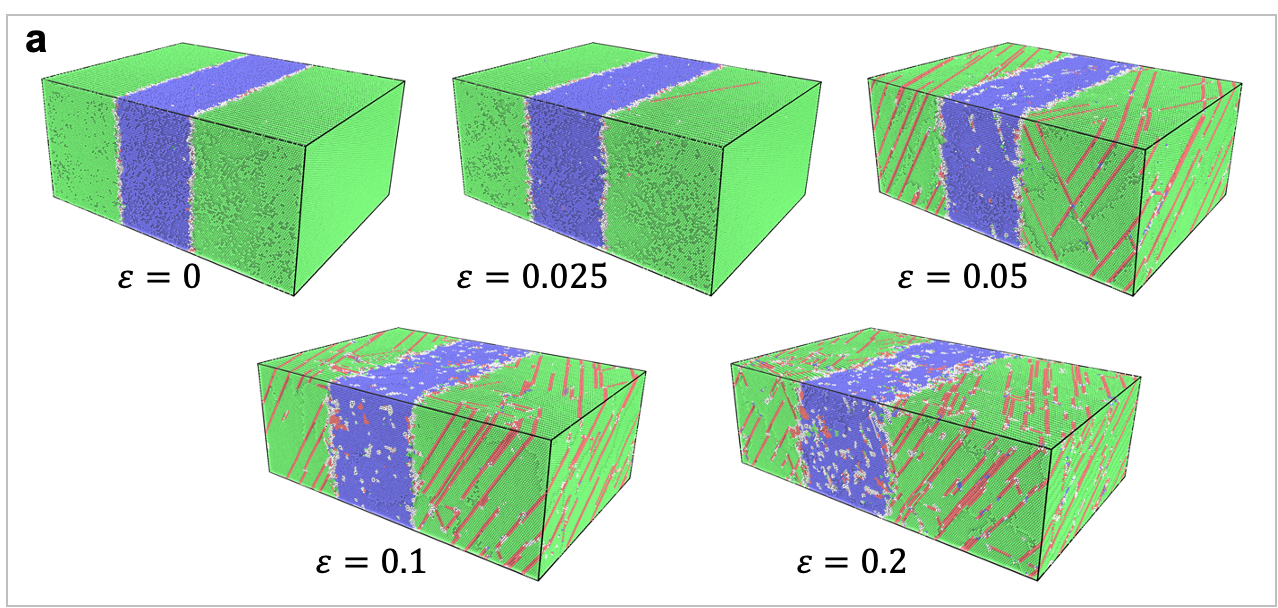  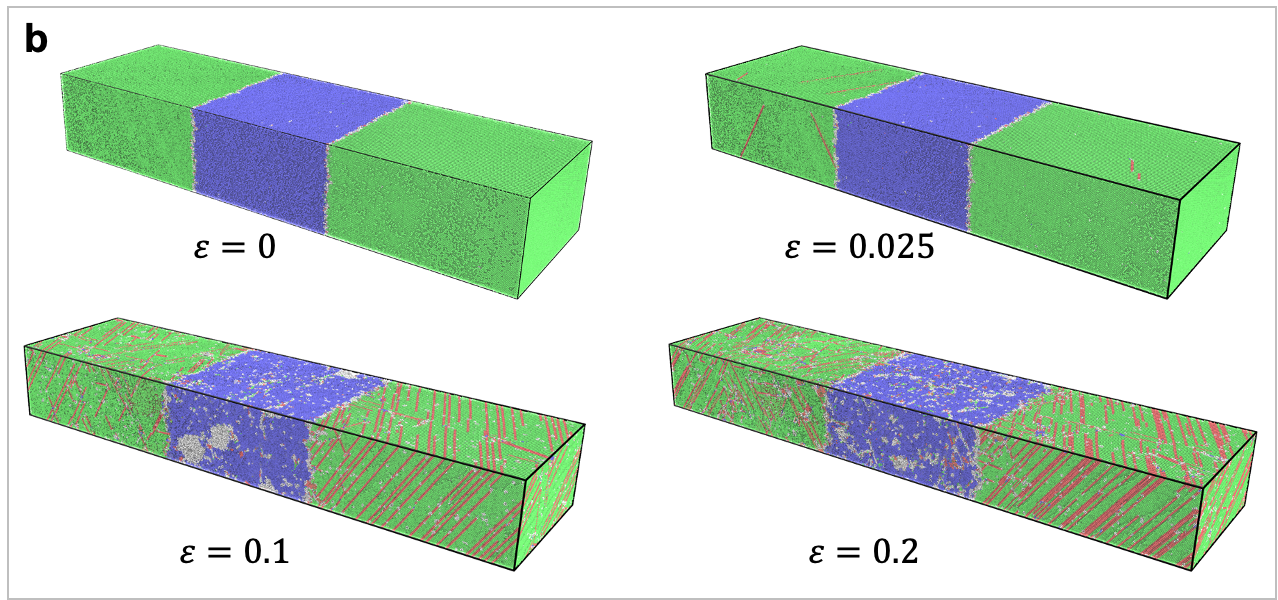 \| \| --- \| \| **Figure S2.** Deformation process of the simulated EHEAs at different strain $\varepsilon$ as a function of the interphase boundary spacings: a) 40 nm and b) 108 nm. The FCC atoms are colored green, BCC atoms are colored blue, stacking fault atoms are colored red, and the amorphous atoms are colored grey. \|  \| **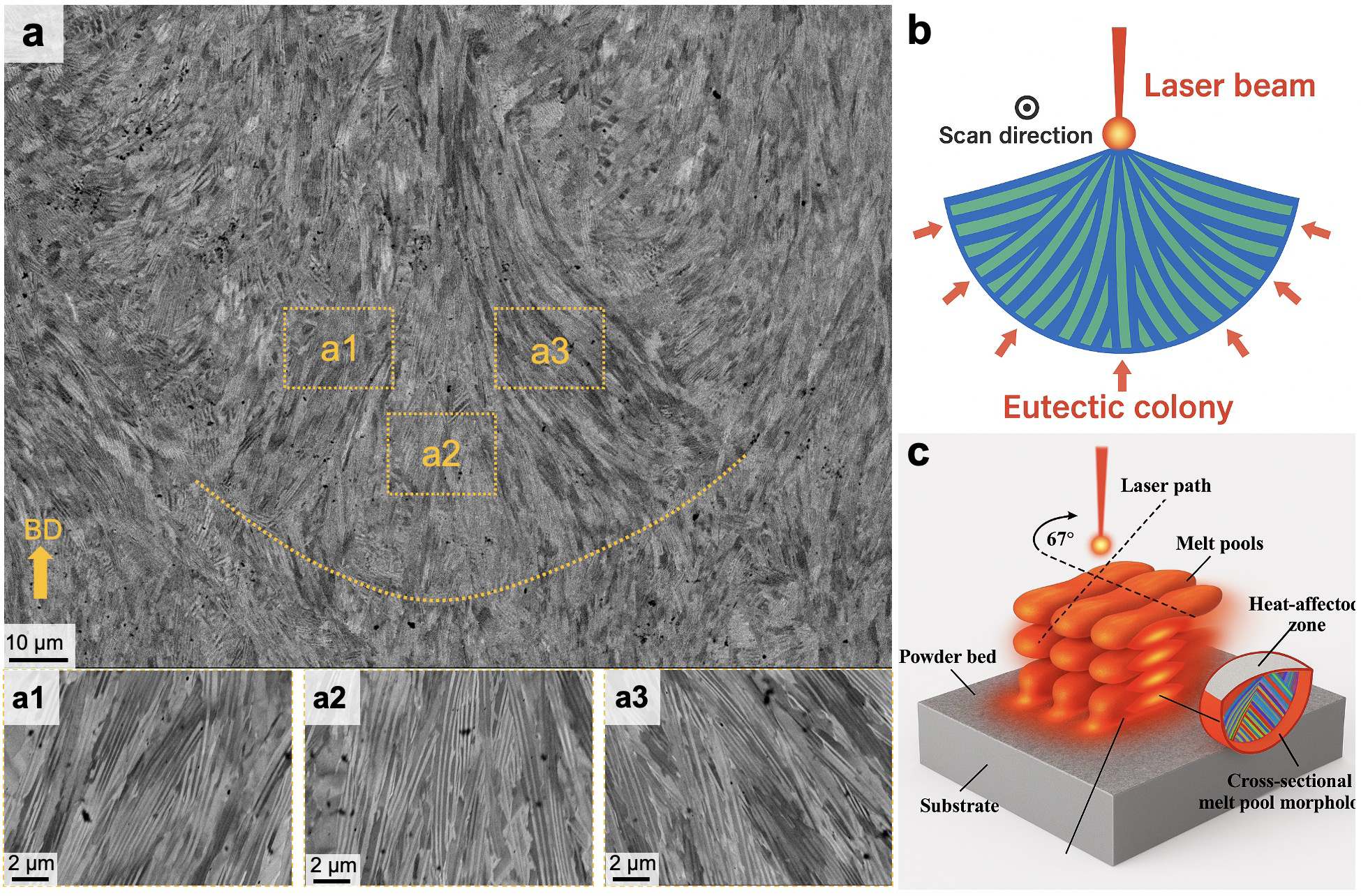** \| \| --- \| \| **Figure S3**. Nearly random orientations in shape and crystallography of lamellar eutectic colonies in as-printed AlCoCrFeNi_2.1_ EHEA. a) Secondary electron image of a cross-section view of the EHEA, showing the overall random shape orientations of eutectic colonies inside a melt pool. The melt pool boundary is outlined by the yellow dashed line. a1–a3 shows magnified images of regions with different shape orientations of lamellar eutectic colonies inside the melt pool. b) Schematic illustration of the nearly random orientations in two-dimensional shape and crystallography of eutectic colonies inside a melt pool. c) Schematic illustration of the nearly random orientations in the three-dimensional shape and crystallography of eutectic colonies after using the 67° rotation scanning strategy. \|   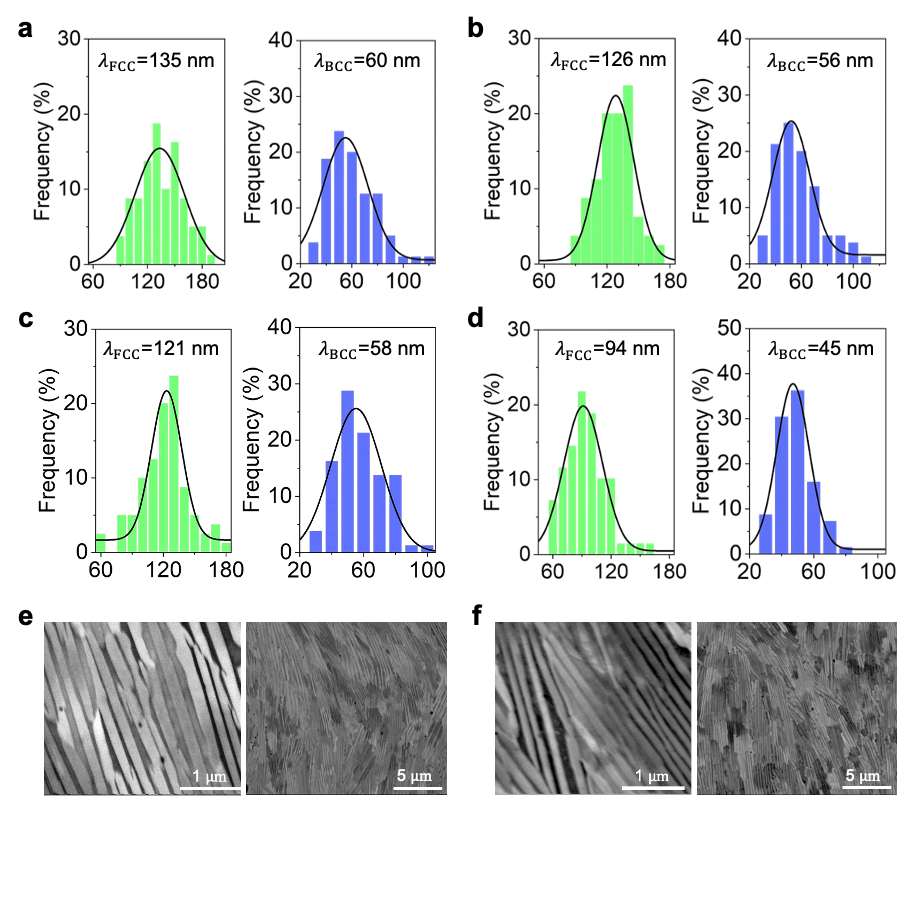 |
| **Figure S4.** Backscatter electron (BSE) SEM images of the lamellar microstructure and FCC/BCC lamellar thickness distribution. a) Interphase boundary spacing of 195 mm; b) Interphase boundary spacing of 182 mm; c) Interphase boundary spacing of 179 mm; d) Interphase boundary spacing of 139 mm; e) BSE image of the nanolamellae with interphase boundary spacing of 195 mm; f) BSE image of the nanolamellae with interphase boundary spacing of 139 mm. |

| 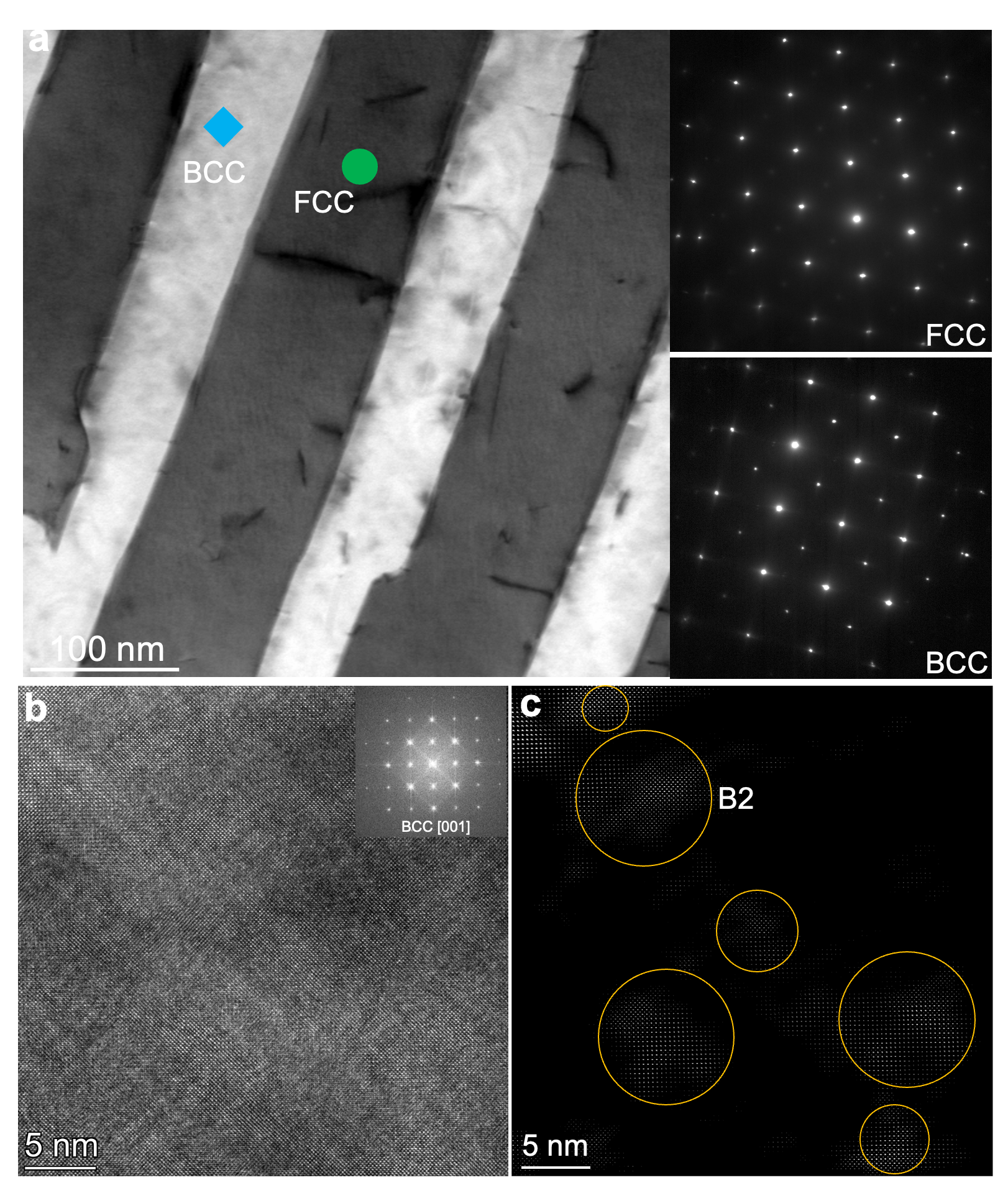 |
| --- |
| **Figure S5**. TEM characterization for microstructure of the as-printed AlCoCrFeNi_2.1_ EHEAs. a) Scanning transmission electron microscopy image of the as-printed AlCoCrFeNi_2.1_ EHEAs with the insets showing SAED patterns from FCC and BCC lamellae tilted to the zone axes [110] and [111], respectively; b) Large-area high-resolution transmission electron microscopy (HRTEM) image with the inset shows the corresponding fast Fourier transform pattern (FFT); c) Inverse FFT image of b) exhibit some regions highlighted by yellow circles, indicative of the presence of B2 precipitates. |
| 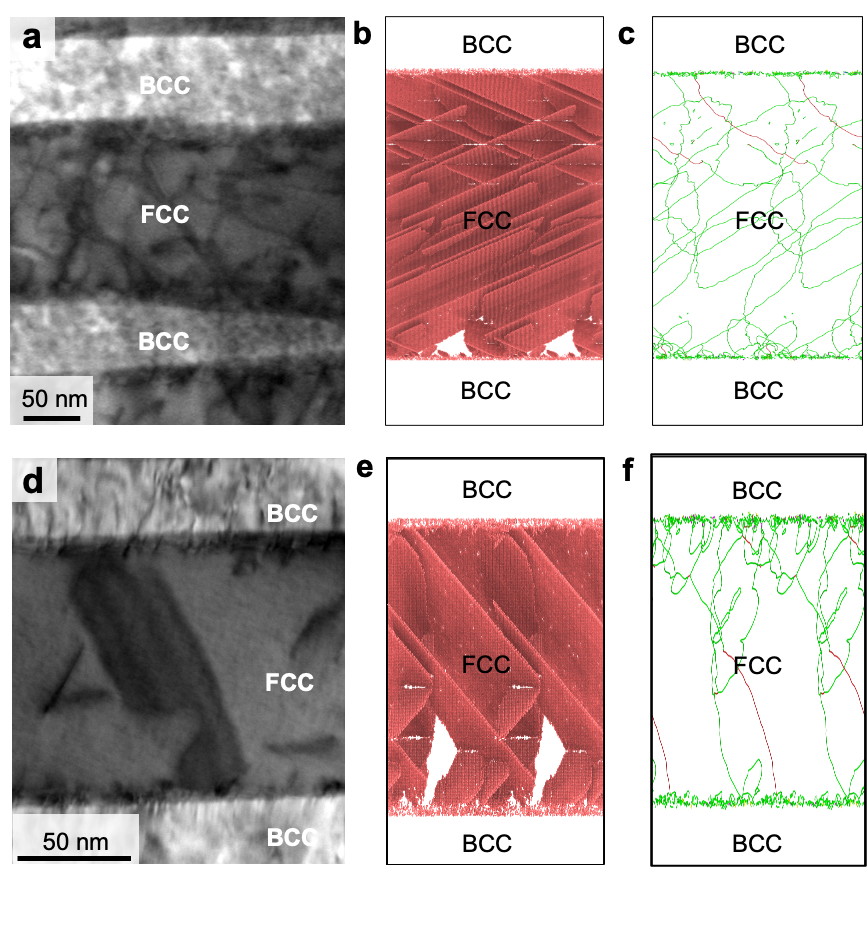 |
| **Figure S6.** Experimental and simulated dislocation behaviors at different interphase boundary spacings. a) Scanning transmission electron microscopy (STEM) image of dislocation multiplications near the interphase boundary. b) Simulated dislocation multiplication near the interphase boundary. Only atoms in stacking faults are shown. c) Corresponding dislocation behavior of b). d) STEM image of stacking faults within the FCC layer. e) Simulated stacking faults during single dislocations glide. f) Corresponding dislocation behavior of e). |

| 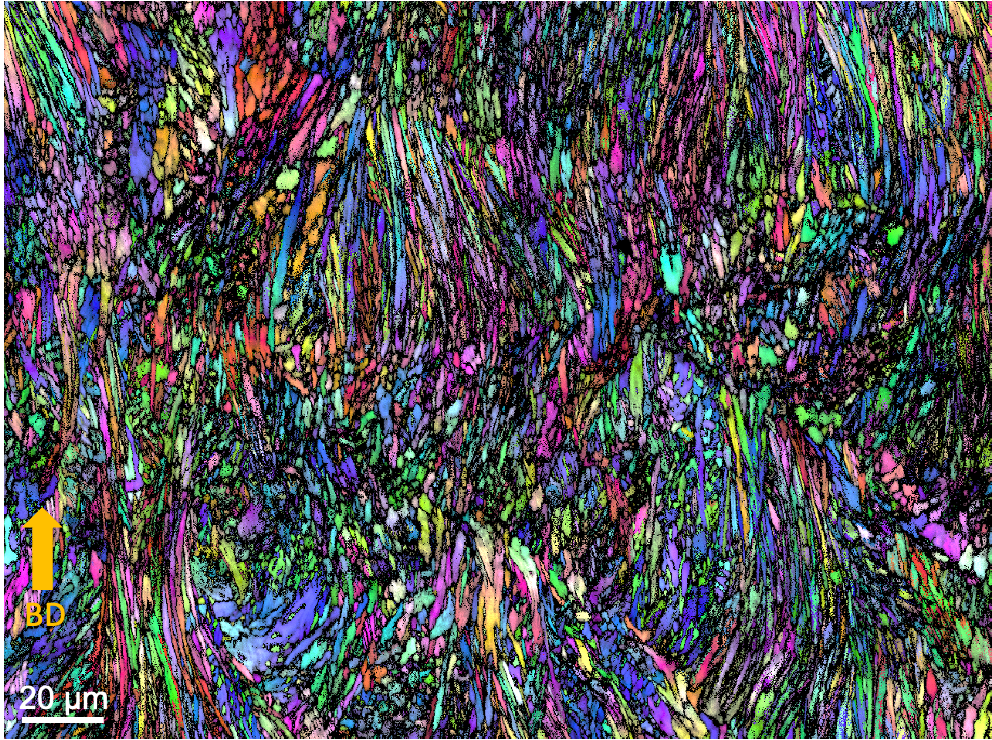 |
| --- |
| **Figure S7**. Inverse pole figure (IPF) color codes with respect to the build direction showing the detailed microstructure. |

| \| 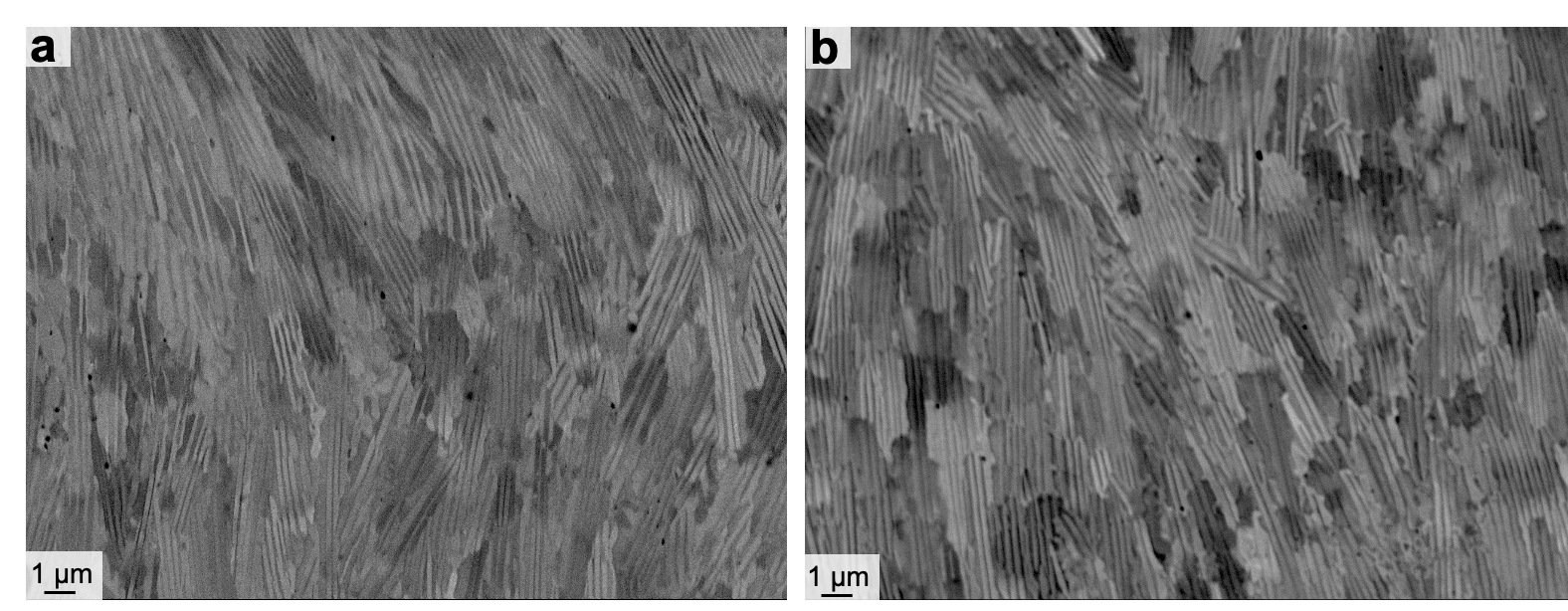 \| \| --- \| \| **Figure S8.** Back-scattered SEM micrographs of the alternating FCC (bright contrast) and BCC (dark contrast) lamellae. a) Sample A; b) Sample D. \| |
| --- | --- | --- |

| 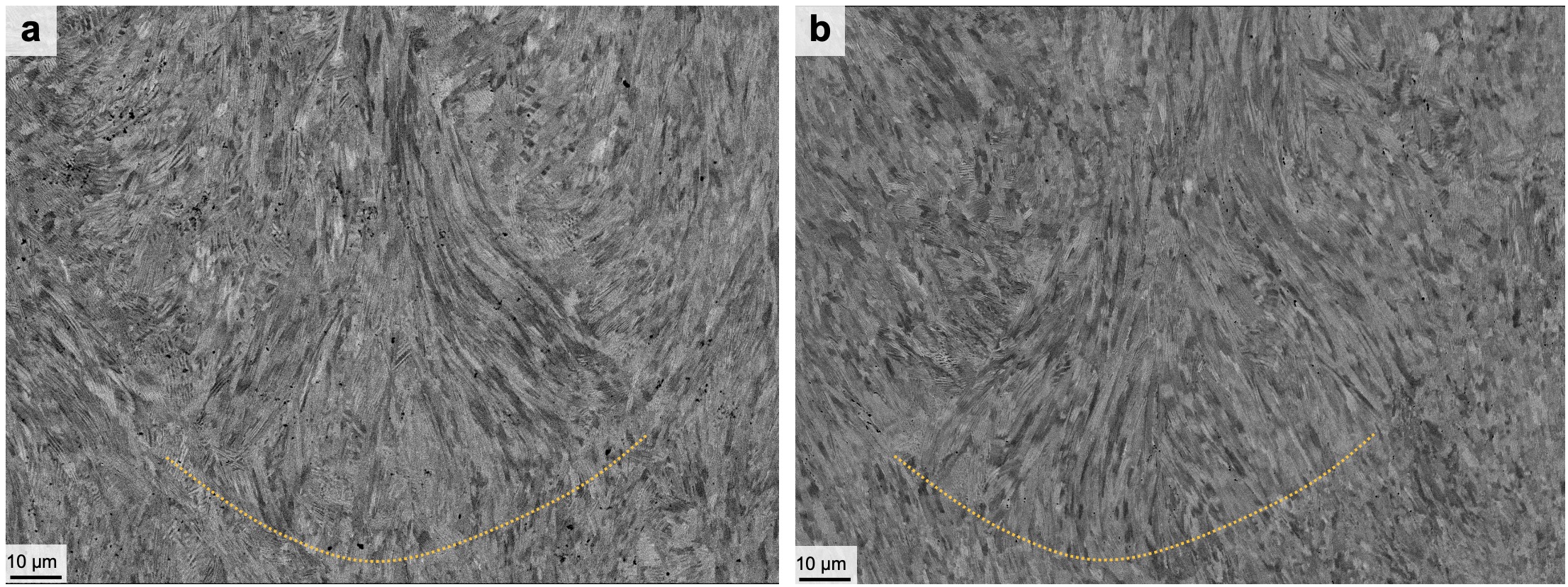 |
| --- |
| **Figure S9.** Back-scattered SEM images of a cross-section view of the as-printed EHEA. a) Sample A; b) Sample D. |

| 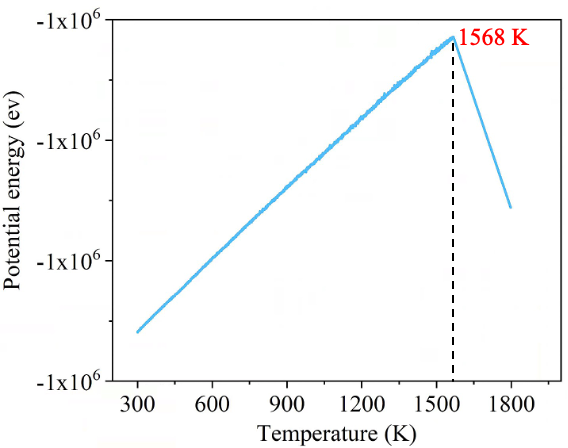 |
| --- |
| **Figure S10.** Simulated temperature dependence of potential energy curves for AlCoCrFeNi_2.1_ EHEAs. The melting temperature is around 1568 K, which coincides with the experimentally measured temperature of 1616 K. |

| 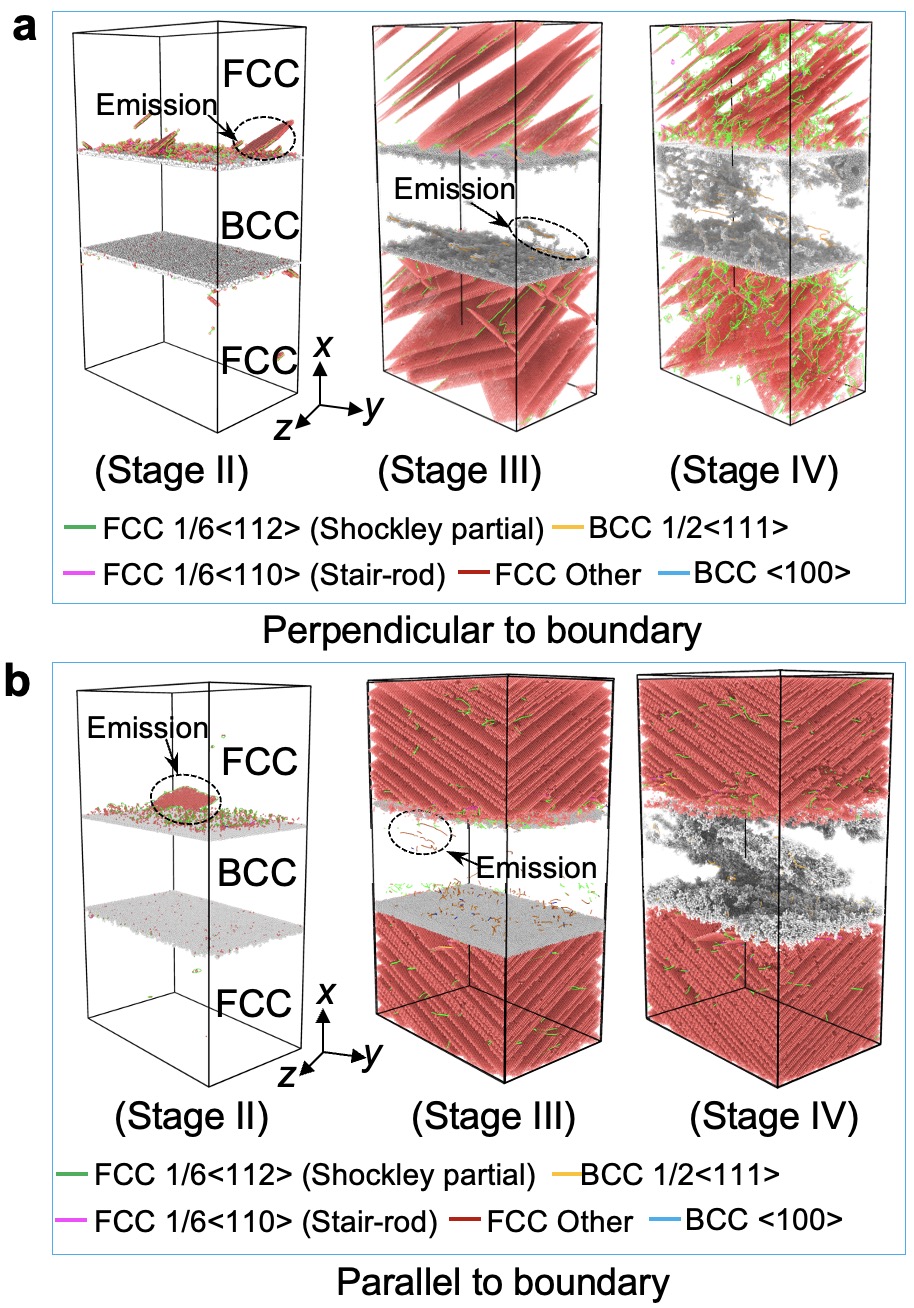 |
| --- |
| **Figure S11**. Atomic deformation of the AlCoCrFeNi_2.1_ EHEA with an interphase boundary spacing of 40 nm. a) Deformation behavior of the EHEA with the tensile axis perpendicular to the interphase boundary; b) Deformation behavior of the EHEA with the tensile axis parallel to the interphase boundary. Both simulation results showed multiplication of SFs connecting boundaries. The SFs are marked by red atoms, and the dislocation lines are colored based on their slip planes. |
| **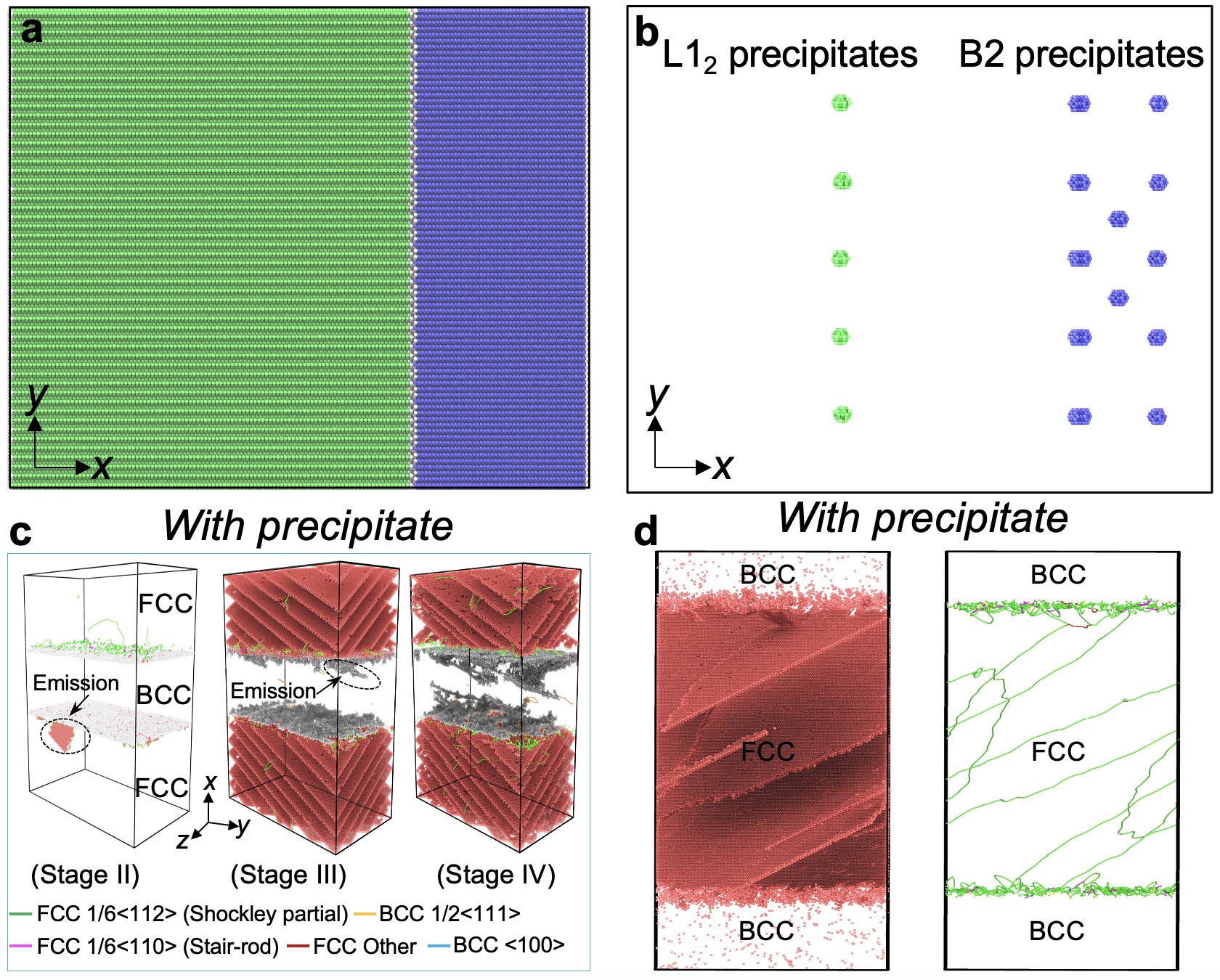** |
| **Figure S12**. Atomic deformation of the AlCoCrFeNi_2.1_ EHEA with L1_2_ and B2 nanoprecipitates (diameter = 1.5 nm) in the FCC and BCC lamellae, respectively. a) Atomistic model of the AlCoCrFeNi_2.1_ EHEA; b) Distributions of L1_2_ (0.5%) and B2 (14%) nanoprecipitates within the FCC and BCC lamellae, respectively; c) Deformation behavior of an EHEA with an interphase boundary spacing of 40 nm; d) Simulated stacking faults during single dislocations glide and corresponding dislocation behavior. |

| 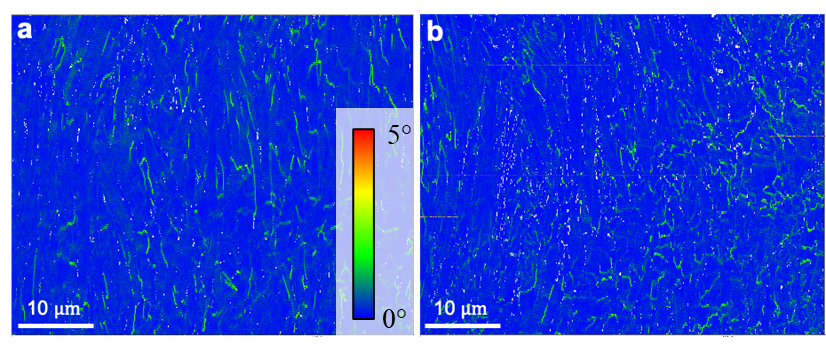 |
| --- |
| **Figure S13.** Kernel average misorientation (KAM) map of the as-printed samples prior to deformation. a) Sample A. b) Sample D. |

| 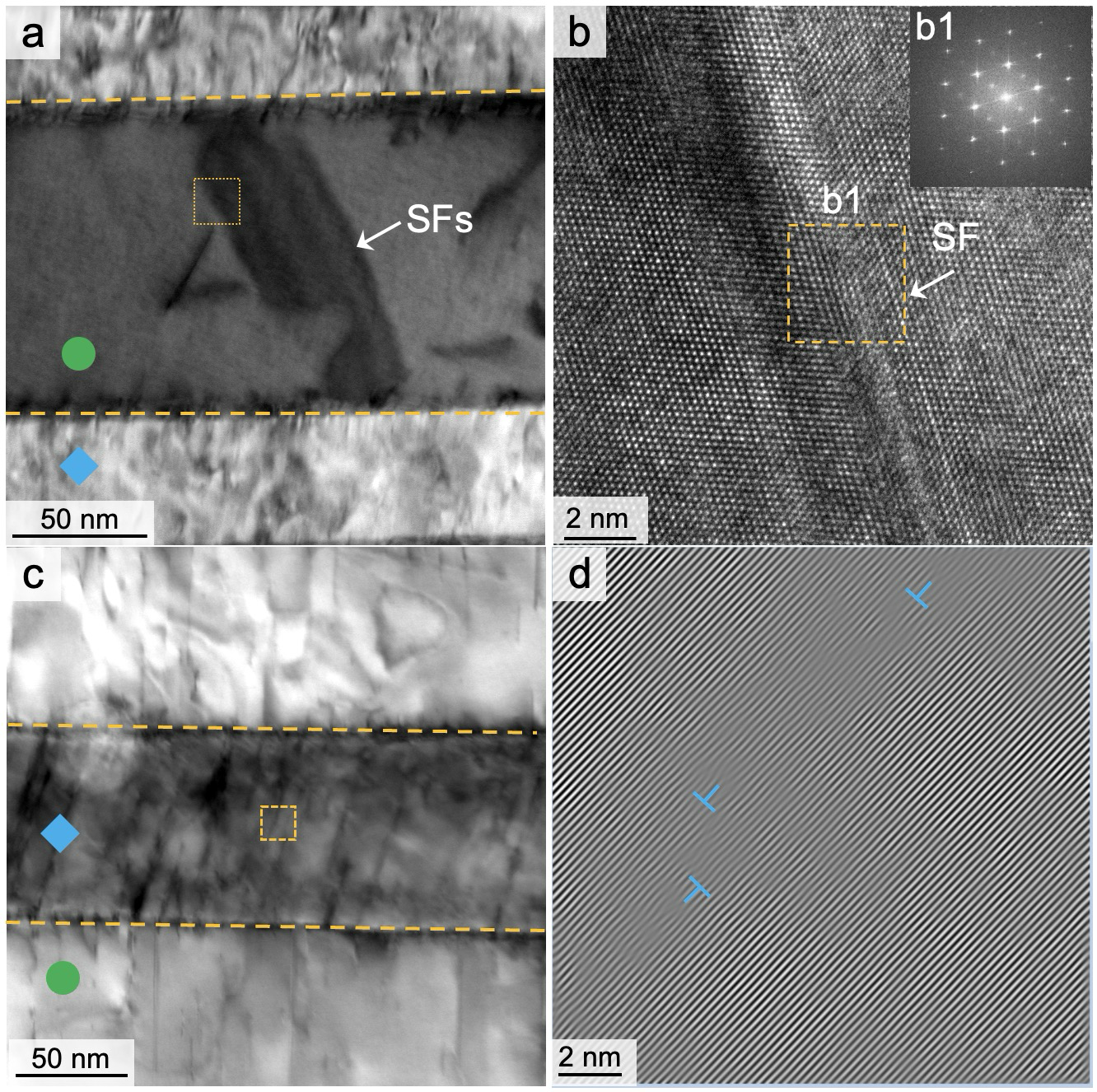 |
| --- |
| **Figure S14**. Bright-field TEM images of as-printed eutectic lamellae. a) Stacking faults connecting two adjacent phase boundaries within the FCC lamellae; b) HRTEM image of the marked area in a) showing the deformation-induced SFs. The inset shows the FFT micrograph of the SF; c) Dislocations in the BCC lamellae. The FCC and BCC phases are marked by green and blue dots, respectively; d) HRTEM image of the marked region in c) showing the BCC dislocations. |

**Table S1.** Summary of the tensile properties of additively manufactured metallic materials in Figure 3e, including ultimate strength $\sigma_{u}$ and uniform elongation $\varepsilon_{u}$.

| **Composition** | **AM method** | $\boldsymbol{\sigma}_{\mathbf{u}}$ **(MPa)** | $\boldsymbol{\varepsilon}_{\mathbf{u}}$ **(%)** | **Symbol** |
| --- | --- | --- | --- | --- |
| AlCoCrFeNi_2.1_ (current work) | Laser powder bed fusion | 1800 | 8.69 |  |
|  |  | 1774 | 9.61 |  |
|  |  | 1747 | 12.3 |  |
|  |  | 1666 | 12.63 |  |
| 17–4PH stainless steels ^[6]^ | Laser powder bed fusion | 1417 | 11.7 |  |
|  |  | 1358 | 6.5 |  |
|  |  | 1319 | 4.8 |  |
|  |  | 1188 | 3.5 |  |
|  |  | 1444 | 2.9 |  |
|  |  | 1172 | 4.8 |  |
|  |  | 1017 | 7.7 |  |
| 15–5PH stainless steels ^[7]^ | Laser powder bed fusion | 1450 | 9 |  |
|  |  | 1467 | 9 |  |
| 4041 steels ^[8]^ | Laser powder bed fusion | 1280 | 7 |  |
|  |  | 1325 | 5 |  |
| HY100 steels ^[9]^ | Laser powder bed fusion | 1200 | 7 |  |
| Maraging steels ^[10]^ | Laser powder bed fusion | 1118 | 7 |  |
|  |  | 1410 | 2 |  |
| AF9628 martensitic steels ^[11]^ | Laser powder bed fusion | 1400 | 7.2 |  |
|  |  | 1310 | 5.8 |  |
|  |  | 1410 | 6.3 |  |
|  |  | 1340 | 4.4 |  |
|  |  | 1430 | 7 |  |
|  |  | 1320 | 6.6 |  |
|  |  | 1420 | 6.1 |  |
|  |  | 1380 | 6.4 |  |
| Damascus steels ^[12]^ | Laser directed energy deposition | 1314 | 8.8 |  |
|  |  | 1431 | 9 |  |
|  |  | 1313 | 9 |  |
| H13 tool steels ^[13]^ | Laser powder bed fusion | 1712 | 4.1 |  |
|  |  | 1620 | 4.1 |  |
|  |  | 1965 | 3.7 |  |
| Ti–6Al–4V ^[14]^ | Laser powder bed fusion | 1420 | 4 |  |
| Ti–6Al–2Sn–4Zr–6Mo ^[15]^ | Laser powder bed fusion | 1125 | 10.9 |  |
|  |  | 1146 | 10.5 |  |
| Ti–6.5Al–3.5Mo–1.5Zr–0.3Si ^[16]^ | Laser directed energy deposition | 1103 | 14 |  |
| Ti–0.4Ni ^[17]^ | Laser directed energy deposition | 1073 | 5.5 |  |
| Ti–6.5Cu ^[18]^ | Laser powder bed fusion | 1120 | 6.8 |  |
| Ti–8.5Cu ^[18]^ | Laser directed energy deposition | 1180 | 2.1 |  |

**Table S1.** Continued.

| **Composition** | **AM method** | $\boldsymbol{\sigma}_{\mathbf{u}}$ **(MPa)** | $\boldsymbol{\varepsilon}_{\mathbf{u}}$ **(%)** | **Symbol** |
| --- | --- | --- | --- | --- |
| Inconel 718 ^[19–21]^ | Laser directed energy deposition | 1333 | 8 |  |
|  |  | 1221 | 16 |  |
|  |  | 1194 | 19 |  |
|  | Laser powder bed fusion | 1126 | 19 |  |
|  |  | 1371 | 10 |  |
|  |  | 1371 | 12.3 |  |
|  | Selective electron beam melting | 1138 | 15 |  |
|  |  | 1061 | 11.5 |  |
|  |  | 1266 | 15 |  |
|  |  | 1240 | 17 |  |
| CoCrW ^[22]^ | Laser powder bed fusion | 1158 | 9.8 |  |
| Co_1.5_CrFeNi_1.5_Ti_0.5_Mo_0.1_ ^[23]^ | Selective electron beam melting | 1323 | 17 |  |
| Ni_2.1_CoCrFeNb_0.2_ ^[24]^ | Laser directed energy deposition | 1127 | 15.3 |  |
|  |  | 1045 | 12.6 |  |
| Ni_30_Co_30_Cr_10_Fe_10_Al_18_W_1_Mo_1_ ^[25]^ | Laser powder bed fusion | 1402 | 18 |  |
| Fe_29.32_Co_28.72_Ni_28.64_Al_6.78_ Ti_6.56_ ^[26]^ | Laser powder bed fusion | 1381 | 6.1 |  |
| CrMnFeCoNi + 5 wt% TiN ^[27]^ | Laser powder bed fusion | 1036 | 12 |  |
| CrMnFeCoNi + 12 wt% TiN ^[28]^ | Laser powder bed fusion | 1100 | 8 |  |
| Cr_36_Co_32_Ni_32_ + 3 wt% TiC ^[29]^ | Laser powder bed fusion | 1340 | 15.6 |  |
| Ni_40_Co_20_Fe_10_Cr_10_Al_18_W_2_^[30]^ | Laser powder bed fusion | 1640 | 16.5 |  |
| AlCoCrFeNi_2.1_^[1]^ | Laser powder bed fusion | 1640 | 13.5 |  |
| AlCoCrFeNi_2.1_^[1]^ | Laser powder bed fusion | 1517 | 16.6 |  |
| AlCoCrFeNi_2.1_^[31]^ | Laser powder bed fusion | 1220 | 20 |  |
| AlCoCrFeNi_2.1_^[32]^ | Laser powder bed fusion | 1150 | 20 |  |
| AlCoCrFeNi_2.1_^[33]^ | Laser powder bed fusion | 1480 | 6 |  |
| AlCoCrFeNi_2.1_^[34]^ | Laser powder bed fusion | 1410 | 16 |  |
| N_i30_Co_30_Cr_10_Fe_10_Al_18_W_2_^[35]^ | Laser powder bed fusion | 1549 | 4.7 |  |
| AlCoCrFeNi_2.1_^[36]^ | Laser powder bed fusion | 1402 | 15 |  |
| AlCoCrFeNi_2.1_^[37]^ | Laser powder bed fusion | 1364 | 16.4 |  |
| AlCoCrFeNi_2.1_^[36]^ | Laser powder bed fusion | 1402 | 15 |  |
| **Table S1.** Continued. | | | | |
| AlCoCrFeNi_2.1_^[38]^ | Laser powder bed fusion | 1436 | 17.5 |  |
| AlCoCrFeNi_2.1_^[39]^ | Laser powder bed fusion | 1508 | 10.9 |  |
| AlCoCrFeNi_2.1_^[37]^ | Laser powder bed fusion | 1421 | 10 |  |
| AlCoCrFeNi_2.1_^[40]^ | Laser powder bed fusion | 1418 | 3.19 |  |
| AlCoCrFeNi_2.1_^[40]^ | Laser powder bed fusion | 1242 | 10.2 |  |
| Al_0.7_CoCrFeNi_2.4_^[41]^ | Laser powder bed fusion | 1300 | 20 |  |
| AlCoCrFeNi_2.1_^[42]^ | Laser powder bed fusion | 1403 | 4.4 |  |
| AlCoCrFeNi_2.1_^[43]^ | Laser powder bed fusion | 1430 | 18.5 |  |
| Boron-doped AlCoCrFeNi_2.1_^[44]^ | Laser powder bed fusion | 1539 | 8.2 |  |
| AlCoCrFeNi_2.1_^[45]^ | Laser powder bed fusion | 1495 | 12 |  |
| AlCoCrFeNi_2.1_^[46]^ | Laser powder bed fusion | 1263 | 15.3 |  |
| (CoCrNi)_83_Al_17_^[47]^ | Laser powder bed fusion | 1500 | 4.5 |  |

**Table S2.** Summary of the ultimate tensile strength $\sigma_{u}$ of as-printed AlCoCrFeNi_2.1_ as a function of interphase boundary spacing *S* in Figure 3f. These data are utilized for fitting the Hall–Petch relationship to obtain the upper bound of the ultimate tensile strength of the as-printed AlCoCrFeNi_2.1_ eutectic high-entropy alloy (EHEAs). The additively manufactured AlCoCrFeNi_2.1_ EHEAs with post-heat treatment were not included in our work. The post-heat treatment is able to generate precipitates, such as Co–Cr–Fe rich needle-like precipitates and Cr-rich spherical nano-precipitates,^[1]^ contributing to additional precipitation strengthening. This will result in an underestimation of the ultimate tensile strength predicted by the Hall–Petch relationship.

| **Additive manufacturing method** | $\boldsymbol{\sigma}_{\boldsymbol{0.2}}$ **(MPa)** | $\boldsymbol{\sigma}_{\mathbf{u}}$ **(MPa)** | $\boldsymbol{S}$ **(nm)** | **Ref.** | **Symbol** |
| --- | --- | --- | --- | --- | --- |
| Laser powder bed fusion | 1442 | 1800 | 179 | Current work | 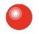 |
|  | 1395 | 1774 | 182 |  |  |
|  | 1375 | 1747 | 195 |  |  |
|  | 1376 | 1666 | 142 |  |  |
| Laser powder bed fusion | 1333 | 1640 | 215 | ^[1]^ | 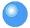 |
|  | 1252 | 1517 | 229 |  |  |
| Laser powder bed fusion | 1116 | 1436 | 300 | ^[38]^ |  |
| Laser powder bed fusion | 1199 | 1421 | 298 | ^[37]^ |  |
| Laser powder bed fusion | 1040 | 1200 | 500 | ^[31]^ |  |
| Laser powder bed fusion | 1210 | 1414 | 244 | ^[34]^ |  |
| Laser powder bed fusion | 1259 | 1418 | 300 | ^[40]^ |  |

**Table S3.** Average phase fraction of the FCC and BCC phases based on experimental measurement.

| **Samples** | **A** | **B** | **C** | **D** |
| --- | --- | --- | --- | --- |
| Tensile strength (MPa) | 1747 | 1774 | 1800 | 1666 |
| FCC volume fraction (%) | 67.45 | 65.97 | 62.45 | 64.39 |
| BCC volume fraction (%) | 32.55 | 34.03 | 37.55 | 35.61 |

**Table S4.** Chemical composition of the EHEA powder.

| Element | Al | Co | Cr | Fe | O | Ni |
| --- | --- | --- | --- | --- | --- | --- |
| Percentage (wt%) | 8.45 | 18.56 | 16.44 | 17.89 | 0.018 | Bal. |

**Table S5.** Process parameters for the LPBF additive manufacturing.

| Processing parameters | Sample A | Sample B | Sample C | Sample D (melting × 2) |
| --- | --- | --- | --- | --- |
| Laser powder (W) | 350 | 350 | 350 | 350 |
| Scan speed (mm/s) | 1000 | 1000 | 800 | 1000 |
| Layer thickness ($\mu$m) | 40 | 40 | 40 | 40 |
| Hatch spacing ($\mu$m) | 80 | 100 | 100 | 80 |
| Rotation angle ($^{\circ}$) | 67 | 67 | 67 | 67 |

**Table S6.** Measured relative density for the LPBF printed specimens.

| Samples | A | B | C | D |
| --- | --- | --- | --- | --- |
| Density (%) | 99.63 ± 0.06 | 99.50 ± 0.04 | 99.56 ± 0.2 | 99.7 ± 0.03 |

**Table S7.** Composition (in at%) of the FCC and BCC phases used in simulation based on previous experiment.^[1]^

| **Phase** | **Al** | **Co** | **Cr** | **Fe** | **Ni** |
| --- | --- | --- | --- | --- | --- |
| FCC | 12.8 | 17.4 | 17.9 | 18.6 | 33.3 |
| BCC | 21.3 | 15.2 | 14.2 | 15.2 | 33.8 |

**Table S8.** Hybrid atomic potentials for AlCoCrFeNi_2.1_ EHEA. With multiple pair styles in one simulation,

the Al–Co–Cr–Fe–Ni potential^[48]^ is placed first, followed by the Al–Co–Cr potential^[49,50]^, and the Al–Ni potential is placed at the end.^[51]^

| **Elements** | **References** |
| --- | --- |
| Al–Co–Cr–Fe–Ni | ^[48]^ |
| Al–Co–Cr | ^[49,50]^ |
| Al–Ni | ^[51]^ |

**Table S9.** Simulated lattice constants of the AlCoCrFeNi_2.1_ EHEA structure as compared to experimental measurements (in brackets)

| **Lattice parameter (Å)** | | **Lattice mismatch (%)** |
| --- | --- | --- |
| **FCC** | **BCC** |  |
| 3.452 (3.596 ^[1]^) | 2.746 (2.868 ^[1]^) | 2.6 (2.3 ^[1]^) |

**Table S10.** DFT calculation of cohesive energy for elemental pairs in binary L1_2_ AB_3_ structures.

|  | **Al_3_** | **Co_3_** | **Cr_3_** | **Fe_3_** | **Ni_3_** |
| --- | --- | --- | --- | --- | --- |
| **Al** | 3.430 | 4.841 | 3.746 | 4.702 | 4.878 |
| **Co** | 4.067 | 5.124 | 4.153 | 4.793 | 4.736 |
| **Cr** | 3.552 | 4.818 | 3.677 | 4.561 | 3.970 |
| **Fe** | 3.901 | 4.880 | 4.018 | 4.698 | 4.889 |
| **Ni** | 3.985 | 5.047 | 4.104 | 4.797 | 4.783 |

**Table S11.** Atomistic calculation of cohesive energy for elemental pairs in binary L1_2_ AB_3_ structures using the hybrid interatomic potentials.

|  | **Al_3_** | **Co_3_** | **Fe_3_** | **Ni_3_** | **Cr_3_** |
| --- | --- | --- | --- | --- | --- |
| **Al** | 3.360 | 4.508 | 4.550 | 4.582 | 4.655 |
| **Co** | 4.027 | 4.357 | 4.509 | 4.400 | 4.129 |
| **Fe** | 3.649 | 4.363 | 4.353 | 4.392 | 4.206 |
| **Ni** | 3.893 | 4.381 | 4.599 | 4.450 | 4.224 |
| **Cr** | 3.518 | 4.350 | 4.273 | 4.345 | 3.910 |

**Table S12.** DFT calculation of cohesive energy for elemental pairs in binary B2 AB structures.

|  | **Al** | **Co** | **Cr** | **Fe** | **Ni** |
| --- | --- | --- | --- | --- | --- |
| **Al** | 3.338 | 4.885 | 3.782 | 4.476 | 4.770 |
| **Co** | 4.885 | 4.697 | 4.434 | 5.054 | 4.957 |
| **Cr** | 3.782 | 4.434 | 4.065 | 4.275 | 4.169 |
| **Fe** | 4.476 | 5.054 | 4.275 | 4.386 | 4.443 |
| **Ni** | 4.770 | 4.957 | 4.169 | 4.443 | 4.675 |

**Table S13.** Atomistic calculation of cohesive energy for elemental pairs in binary B2 AB structures.

|  | **Al** | **Co** | **Cr** | **Fe** | **Ni** |
| --- | --- | --- | --- | --- | --- |
| **Al** | 3.000 | 4.973 | 4.308 | 5.034 | 4.485 |
| **Co** | 4.973 | 4.359 | 4.333 | 5.203 | 4.304 |
| **Fe** | 5.034 | 5.203 | 4.205 | 4.301 | 4.301 |
| **Ni** | 4.485 | 4.304 | 4.207 | 4.301 | 4.336 |
| **Cr** | 4.308 | 4.333 | 4.100 | 4.205 | 4.207 |

**Table S14.** Elastic constants and bulk modulus of FCC and BCC phases predicted by atomistic simulations. The values in the brackets are obtained from previous experimental results.^[1]^

|  | **FCC (GPa)** | **BCC (GPa)** |
| --- | --- | --- |
| **C_11_** | 255(258.7) | 254.7(282.4) |
| **C_12_** | 213(177.5) | 210.8(259.8) |
| **C_44_** | 173(124.8) | 188.5(131.9) |
| **Bulk modulus** | 256.5 (225.0) | |

**Table S15.** Measured composition (in at%) of the FCC and BCC phases measured by TEM–EDS.

| **Phase** | **Al** | **Co** | **Cr** | **Fe** | **Ni** |
| --- | --- | --- | --- | --- | --- |
| FCC | 12.4 ± 1.1 | 17.2 ± 0.6 | 19.4 ± 0.1 | 17.2 ± 0.6 | 33.7 ± 0.7 |
| BCC | 27.3 ± 0.3 | 13.2 ± 0.2 | 12.7 ± 0.3 | 11.3 ± 0.1 | 35.5 ± 0.1 |

**Table S16.** Calculated dislocation density of the as-printed samples prior to deformation.

| Samples | A | B | C | D |
| --- | --- | --- | --- | --- |
| Tensile strength (MPa) | 1747 | 1774 | 1800 | 1666 |
| Dislocation density (10^14^ m^–2^) | 3.19 | 4.54 | 4.83 | 3.42 |

**Table S17.** The total enhancement of yield strength $\sigma_{y}$ from friction stress (95 MPa), lamellar boundary $\sigma_{\mathrm{LB}}$, pre-existing dislocations $\sigma_{\rho}$, and nanoprecipitates $\sigma_{S}$ in the AlCoCrFeNi_2.1_ EHEAs.

| FCC Thickness (nm) | Predicted $\sigma_{y}$ (MPa) | Measured $\sigma_{y}$ (MPa) |
| --- | --- | --- |
| 135 | 1543 | 1438 |
| 126 | 1492 | 1405 |
| 121 | 1381 | 1398 |

**SI References**

[1] J. Ren, Y. Zhang, D. Zhao, Y. Chen, S. Guan, Y. Liu, L. Liu, S. Peng, F. Kong, J. D. Poplawsky, *Nature* **2022**, *608*, 62.

[2] X. Gao, Y. Lu, B. Zhang, N. Liang, G. Wu, G. Sha, J. Liu, Y. Zhao, *Acta Materialia* **2017**, *141*, 59.

[3] T. Xiong, S. Zheng, J. Pang, X. Ma, *Scripta Materialia* **2020**, *186*, 336.

[4] X. Wang, Q. Zhang, X. Pan, X. Chen, J. Eckert, C. Qiu, *Materials Science and Engineering: A* **2024**, *913*, 147060.

[5] A. Jarlöv, Z. Zhu, W. Ji, S. Gao, Z. Hu, P. Vivegananthan, Y. Tian, D. R. Kripalani, H. Fan, H. L. Seet, *Materials Science and Engineering: R: Reports* **2024**, *161*, 100834.

[6] T. LeBrun, T. Nakamoto, K. Horikawa, H. Kobayashi, *Materials & Design* **2015**, *81*, 44.

[7] H. K. Rafi, T. L. Starr, B. E. Stucker, *Int J Adv Manuf Technol* **2013**, *69*, 1299.

[8] J. Damon, R. Koch, D. Kaiser, G. Graf, S. Dietrich, V. Schulze, *Additive Manufacturing* **2019**, *28*, 275.

[9] J. J. S. Dilip, G. J. Ram, T. L. Starr, B. Stucker, *Additive Manufacturing* **2017**, *13*, 49.

[10] X. Xu, S. Ganguly, J. Ding, S. Guo, S. Williams, F. Martina, *Materials Characterization* **2018**, *143*, 152.

[11] R. Seede, D. Shoukr, B. Zhang, A. Whitt, S. Gibbons, P. Flater, A. Elwany, R. Arroyave, I. Karaman, *Acta Materialia* **2020**, *186*, 199.

[12] P. Kürnsteiner, M. B. Wilms, A. Weisheit, B. Gault, E. A. Jägle, D. Raabe, *Nature* **2020**, *582*, 515.

[13] R. Mertens, B. Vrancken, N. Holmstock, Y. Kinds, J.-P. Kruth, J. Van Humbeeck, *Physics Procedia* **2016**, *83*, 882.

[14] T. Voisin, N. P. Calta, S. A. Khairallah, J.-B. Forien, L. Balogh, R. W. Cunningham, A. D. Rollett, Y. M. Wang, *Materials & Design* **2018**, *158*, 113.

[15] A. Carrozza, A. Aversa, P. Fino, M. Lombardi, *Journal of Alloys and Compounds* **2021**, *870*, 159329.

[16] Y. Zhu, J. Li, X. Tian, H. Wang, D. Liu, *Materials Science and Engineering: A* **2014**, *607*, 427.

[17] Z. Xiong, X. Pang, S. Liu, Z. Li, R. D. K. Misra, *Scripta Materialia* **2021**, *195*, 113727.

[18] D. Zhang, D. Qiu, M. A. Gibson, Y. Zheng, H. L. Fraser, D. H. StJohn, M. A. Easton, *Nature* **2019**, *576*, 91.

[19] H. Qi, M. Azer, A. Ritter, *Metall Mater Trans A* **2009**, *40*, 2410.

[20] D. Zhang, W. Niu, X. Cao, Z. Liu, *Materials Science and Engineering: A* **2015**, *644*, 32.

[21] M. M. Kirka, F. Medina, R. Dehoff, A. Okello, *Materials Science and Engineering: A* **2017**, *680*, 338.

[22] Y. Lu, S. Wu, Y. Gan, J. Li, C. Zhao, D. Zhuo, J. Lin, *Materials Science and Engineering: C* **2015**, *49*, 517.

[23] T. Fujieda, H. Shiratori, K. Kuwabara, M. Hirota, T. Kato, K. Yamanaka, Y. Koizumi, A. Chiba, S. Watanabe, *Materials Letters* **2017**, *189*, 148.

[24] K. Zhou, Z. Wang, F. He, S. Liu, J. Li, J. Kai, J. Wang, *Additive Manufacturing* **2020**, *35*, 101410.

[25] F. Yang, L. Wang, Z. Wang, Q. Wu, K. Zhou, X. Lin, W. Huang, *Journal of Materials Science & Technology* **2022**, *106*, 128.

[26] L. Xu, Y. Jia, S. Wu, Y. Jia, K. Song, G. Wang, *Scripta Materialia* **2023**, *225*, 115189.

[27] B. Li, L. Zhang, Y. Xu, Z. Liu, B. Qian, F. Xuan, *Powder Technology* **2020**, *360*, 509.

[28] B. Li, B. Qian, Y. Xu, Z. Liu, F. Xuan, *Materials letters* **2019**, *252*, 88.

[29] C. Pan, X. Li, H. Luo, K. Kosiba, S. Qu, C. Yang, D. Zhu, C. Zhang, *Materials Science and Engineering: A* **2022**, *840*, 142923.

[30] J. Ren, M. Wu, C. Li, S. Guan, J. Dong, J.-B. Forien, T. Li, K. S. Shanks, D. Yu, Y. Chen, *Acta Materialia* **2023**, *257*, 119179.

[31] Y. Lu, X. Wu, Z. Fu, Q. Yang, Y. Zhang, Q. Liu, T. Li, Y. Tian, H. Tan, Z. Li, *Journal of Materials Science & Technology* **2022**, *126*, 15.

[32] L. Huang, Y. Sun, N. Chen, H. Luan, G. Le, X. Liu, Y. Ji, Y. Lu, P. K. Liaw, X. Yang, *Materials Science and Engineering: A* **2022**, *830*, 142327.

[33] Y. Guo, H. Su, H. Zhou, Z. Shen, Y. Liu, J. Zhang, L. Liu, H. Fu, *Journal of Materials Science & Technology* **2022**, *111*, 298.

[34] Y. Guo, H. Su, P. Yang, Z. Shen, D. Zhao, Y. Zhao, Y. Liu, H. Zhou, *Additive Manufacturing* **2022**, *60*, 103257.

[35] Y. Yu, Y. Zhao, K. Feng, R. Chen, B. Han, K. Ji, M. Qin, Z. Li, U. Ramamurty, *Materials Science and Engineering: A* **2024**, *918*, 147469.

[36] Y. Guo, H. Su, H. Gao, Z. Shen, P. Yang, Y. Liu, D. Zhao, Z. Zhang, M. Guo, X. Tan, *International Journal of Plasticity* **2024**, *179*, 104050.

[37] T. Yu, G. Zhou, Y. Cheng, F. Hu, T. Jiang, T. Sun, Y. Shen, Y. Zhou, J. Li, *Optics & Laser Technology* **2023**, *163*, 109396.

[38] J. Li, D. Ouyang, J. Li, M. Dang, Q. Wang, M. Wang, C. Cai, Q. Wei, *Materials Science and Engineering: A* **2023**, *887*, 145784.

[39] X. Tang, H. Zhang, Z. Zhu, P. Xue, L. Wu, F. Liu, D. Ni, B. Xiao, Z. Ma, *Journal of Materials Science & Technology* **2023**, *150*, 75.

[40] Z. Geng, C. Chen, R. Li, J. Luo, K. Zhou, *Additive Manufacturing* **2022**, *56*, 102941.

[41] Z. Geng, C. Chen, M. Song, J. Luo, J. Chen, R. Li, K. Zhou, *Journal of Materials Science & Technology* **2024**, *187*, 141.

[42] L. He, S. Wu, A. Dong, H. Tang, D. Du, G. Zhu, B. Sun, W. Yan, *Journal of Materials Science & Technology* **2022**, *117*, 133.

[43] W. Luo, X. Yuan, Z. Zhang, C. Cheng, H. Liu, H. Qiu, X. Cheng, *Journal of Alloys and Compounds* **2025**, *1010*, 178032.

[44] H. Gao, H. Su, Y. Guo, P. Yang, Q. Hu, Z. Shen, H. Jiang, M. Yu, X. Li, Z. Zhang, *Materials Science and Engineering: A* **2025**, *924*, 147770.

[45] Y. Guo, H. Su, H. Gao, Z. Shen, Y. Liu, D. Zhao, P. Yang, Q. Hu, Z. Zhang, *Journal of Materials Science & Technology* **2023**, *163*, 81.

[46] Z. Fu, X. Li, P. Wu, L. Liao, *Optics & Laser Technology* **2025**, *183*, 112364.

[47] M. Zheng, C. Li, Y. Liao, Z. Long, J. Gu, *Scripta Materialia* **2025**, *263*, 116655.

[48] D. Farkas, A. Caro, *Journal of Materials Research* **2020**, *35*, 3031.

[49] X. W. Zhou, R. A. Johnson, H. N. G. Wadley, *Phys. Rev. B* **2004**, *69*, 144113.

[50] Z. Lin, R. A. Johnson, L. V. Zhigilei, *Phys. Rev. B* **2008**, *77*, 214108.

[51] G. P. Purja Pun, Y. Mishin, *Philosophical Magazine* **2009**, *89*, 3245.
